# Flexible, multimodal, electrical-sensing–optical-transmission μfiber-sensors via an on-fiber printed electronics strategy

**DOI:** 10.1093/nsr/nwag250

**Published:** 2026-05-07

**Authors:** Hongyang Wang, Dong Ye, Qingshuang Wu, Longkang Yang, Chenyang Zhao, Dada Wu, Yongqi Guo, Wangyang Xu, Wei He, Zhangyu Xu, Hao Wu, Wenlong Lu, Wei Li, Qizhen Sun, YongAn Huang

**Affiliations:** State Key Laboratory of Intelligent Manufacturing Equipment and Technology, Huazhong University of Science and Technology, Wuhan 430074, China; Research Center for Advanced Electronics Manufacturing, Huazhong University of Science and Technology, Wuhan 430074, China; Flexible Electronics Research Center, Huazhong University of Science and Technology, Wuhan 430074, China; State Key Laboratory of Intelligent Manufacturing Equipment and Technology, Huazhong University of Science and Technology, Wuhan 430074, China; Research Center for Advanced Electronics Manufacturing, Huazhong University of Science and Technology, Wuhan 430074, China; Flexible Electronics Research Center, Huazhong University of Science and Technology, Wuhan 430074, China; State Key Laboratory of Intelligent Manufacturing Equipment and Technology, Huazhong University of Science and Technology, Wuhan 430074, China; Research Center for Advanced Electronics Manufacturing, Huazhong University of Science and Technology, Wuhan 430074, China; Flexible Electronics Research Center, Huazhong University of Science and Technology, Wuhan 430074, China; State Key Laboratory of Intelligent Manufacturing Equipment and Technology, Huazhong University of Science and Technology, Wuhan 430074, China; Research Center for Advanced Electronics Manufacturing, Huazhong University of Science and Technology, Wuhan 430074, China; Flexible Electronics Research Center, Huazhong University of Science and Technology, Wuhan 430074, China; State Key Laboratory of Intelligent Manufacturing Equipment and Technology, Huazhong University of Science and Technology, Wuhan 430074, China; Research Center for Advanced Electronics Manufacturing, Huazhong University of Science and Technology, Wuhan 430074, China; Flexible Electronics Research Center, Huazhong University of Science and Technology, Wuhan 430074, China; State Key Laboratory of Intelligent Manufacturing Equipment and Technology, Huazhong University of Science and Technology, Wuhan 430074, China; Research Center for Advanced Electronics Manufacturing, Huazhong University of Science and Technology, Wuhan 430074, China; Flexible Electronics Research Center, Huazhong University of Science and Technology, Wuhan 430074, China; State Key Laboratory of Intelligent Manufacturing Equipment and Technology, Huazhong University of Science and Technology, Wuhan 430074, China; Research Center for Advanced Electronics Manufacturing, Huazhong University of Science and Technology, Wuhan 430074, China; Flexible Electronics Research Center, Huazhong University of Science and Technology, Wuhan 430074, China; School of Optical and Electronics Information, National Engineering Research Center of Next Generation Internet Access System, Huazhong University of Science and Technology, Wuhan 430074, China; State Key Laboratory of Intelligent Manufacturing Equipment and Technology, Huazhong University of Science and Technology, Wuhan 430074, China; Research Center for Advanced Electronics Manufacturing, Huazhong University of Science and Technology, Wuhan 430074, China; Flexible Electronics Research Center, Huazhong University of Science and Technology, Wuhan 430074, China; State Key Laboratory of Intelligent Manufacturing Equipment and Technology, Huazhong University of Science and Technology, Wuhan 430074, China; Research Center for Advanced Electronics Manufacturing, Huazhong University of Science and Technology, Wuhan 430074, China; Flexible Electronics Research Center, Huazhong University of Science and Technology, Wuhan 430074, China; State Key Laboratory of Intelligent Manufacturing Equipment and Technology, Huazhong University of Science and Technology, Wuhan 430074, China; Research Center for Advanced Electronics Manufacturing, Huazhong University of Science and Technology, Wuhan 430074, China; Flexible Electronics Research Center, Huazhong University of Science and Technology, Wuhan 430074, China; State Key Laboratory of Intelligent Manufacturing Equipment and Technology, Huazhong University of Science and Technology, Wuhan 430074, China; AVIC The First Aircraft Institute, Xi’an 710089, China; School of Optical and Electronics Information, National Engineering Research Center of Next Generation Internet Access System, Huazhong University of Science and Technology, Wuhan 430074, China; State Key Laboratory of Intelligent Manufacturing Equipment and Technology, Huazhong University of Science and Technology, Wuhan 430074, China; Research Center for Advanced Electronics Manufacturing, Huazhong University of Science and Technology, Wuhan 430074, China; Flexible Electronics Research Center, Huazhong University of Science and Technology, Wuhan 430074, China

**Keywords:** flexible electronics, electro-optical conversion, multimodal sensing, electrohydrodynamic printing, fiber electronics

## Abstract

Optical fiber sensing offers inherent advantages in long-distance and interference-free transmission. However, it faces a major challenge in achieving self-decoupling and multimodal detection. Here, inspired by the firefly’s bioluminescent mechanism, we propose a flexible, distributed, multimodal electrical-sensing–optical-transmission fiber sensor (ESOT FiSensor) that can convert diverse electrical sensing signals into optical signals through on-fiber hybrid circuits. The ESOT FiSensor realizes distributed and simultaneous monitoring of four physical parameters, including vibration, pressure, temperature, and strain, through only a single optical fiber, and it can maintain long-distance transmission and strong electromagnetic interference immunity within 0–1000 Hz, far superior to purely electrical sensors. The on-fiber electro-optical circuits were fabricated by combining conformal additive printing and flexible hybrid electronics integration, and the printing technique achieves a resolution of 260 nm directly on submillimeter fibers as fine as human hair (∼60 μm). The performance of the ESOT FiSensor has been validated in three representative scenarios: multimodal sensing under complex environmental conditions, distributed sensing on aircraft skins, and wearable sensing for human-machine interaction. The ESOT FiSensor establishes a powerful and scalable platform for long-distance, multimodal signal perception in complex and dynamic environments. It provides a pathway toward transforming optical fibers from passive communication media into active multimodal distributed sensing networks in the near future.

## Teaser

Inspired by fireflies, a novel sensor has been created that integrates electrical sensing and optical transmission in a single fiber, enabling synchronous, long-distance, and interference-resistant monitoring of multiple physical parameters.

## INTRODUCTION

Optical fiber, as a critical component of modern information infrastructure, supports the dissemination of information across a global network that spans over 100 million kilometers. It enables not only high-speed, long-distance signal transmission but also the sensing of external stimuli through changes in optical properties, providing the backbone for 5G, cloud computing, and the Internet of Things [1,2]. Optical fiber sensors, on the other hand, offer advantages including compact size, lightweight structure, resistance to electromagnetic interference (EMI), and corrosion tolerance [3–7]. These features provide them with a wide range of applications in structural health monitoring [8,9], aerospace [10,11], and environmental surveillance [12–14]. However, conventional optical fiber sensors, including those based on Bragg gratings and distributed sensing mechanisms such as Raman [15], Brillouin [16], or Rayleigh [17] scattering, generally struggle to simultaneously sense and decouple multiple physical quantities [18]. These shortcomings constrain their applicability in complex scenarios that require simultaneous multimodal sensing, intrinsic signal decoupling, and robust long-distance transmission.

To enhance the sensing capabilities of optical fibers, several modification strategies have been explored, typically categorized as in-fiber and near-fiber sensitive structures. The in-fiber strategies integrate sensing elements within the fiber core or cladding, including fiber Bragg gratings with different scattering [19–21], surface plasmon resonance structures [22–26], and functional fiber sensors [27–30]. They can achieve high-precision detection of temperature, strain, and other environmental parameters through wavelength modulation and demodulation algorithms. Nevertheless, they are typically limited to monitoring only one or two quantities simultaneously and require complex interrogation systems, and fiber-based multimodal sensors also fail to provide distributed sensing capability. The near-fiber approach places functional devices adjacent to the fiber, either near the end face or along the sidewall, such as end-face coupling photodiodes [4] and attached external optoelectronic sensors [31]. Although they allow the detection of three parameters, only one channel transmits the optical signal. The other parameters based on electrical detections suffer from noise or distance-related loss. Overall, there are still some restrictions for existing strategies in decoupling physical quantities and achieving robust long-distance transmission. Besides, they mostly depend on complex and costly pulsed lasers, which increase system volume, weight, and cost. Thus, it is quite essential to develop a brand-new electro-optical fiber sensing architecture that integrates multimodal sensing, self-decoupling, and long-distance signal transmission within a single platform.

**Figure 1.**
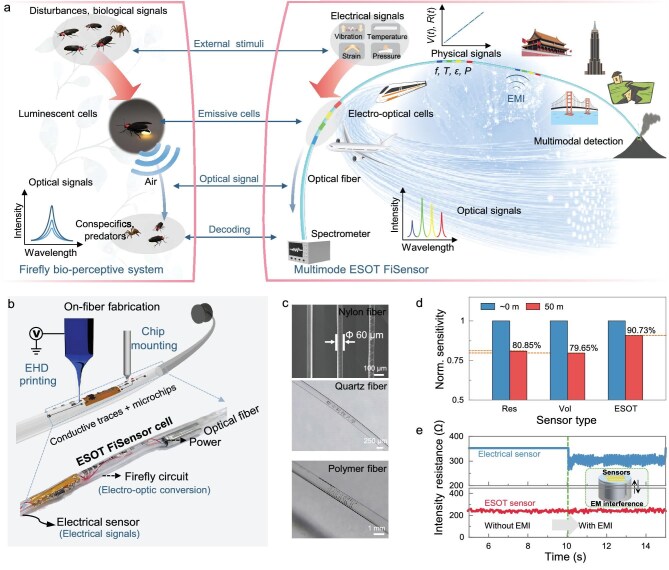
The architecture of the ESOT FiSensor for multimodal physical signal sensing and transmission. (a) The signal-sensing process and application scenarios of the multimodal ESOT FiSensor. (b) Structural composition and fabrication process of the ESOT FiSensor cell. (c) Different types of printed lines on the surface of a nylon fiber with a diameter of 60 μm, and complex printed patterns on quartz fiber and polymer fiber surfaces. (d) Comparison of sensitivity attenuation between the electrical sensor and the ESOT FiSensor after 50 m transmission. (e) Signal performance comparison of the electrical sensor and ESOT FiSensor under electromagnetic interference of 20 Hz.

Fireflies exhibit a natural bioluminescent mechanism that allows them to sense and respond to environmental stimuli [32,33]. As illustrated in Fig. 1a, when stimulated by external cues such as disturbances or biological signals, specialized luminescent cells inside the firefly emit light. This light serves as an information carrier, facilitating communication, mating, or defense by transmitting signals to conspecifics or predators. Inspired by this bioluminescent process, we propose a multimodal electrical-sensing–optical-transmission fiber-sensor (ESOT FiSensor). Specifically, a sensing paradigm is established in which distributed electrical signals are directly converted into wavelength-encoded optical signals within a single fiber. The ESOT FiSensor converts external stimuli into optical signals through an on-fiber electro-optical conversion circuit, emulating the firefly’s bioluminescent behavior. Compared with the firefly’s single fluorescence transmission through air, the fiber-based approach enables longer-distance and multimodal signal transmission through a polymer optical fiber. The proposed ESOT FiSensor system enables multimodal monitoring of vibration, pressure, temperature, and strain simultaneously. For example, the vibration sensing sensitivity reaches 245.7 a.u./mm. This demonstrates a versatile optical fiber-based sensing platform with capabilities in both multimodal detection and signal decoupling. Lateral coupling between the μLEDs and the optical fiber is achieved by structural optimization that ensures effective light injection. Compared with a 300-μm-diameter fiber, the optimized structure reduces optical confinement loss (CL) by 96.3%, confirmed by both systematic simulations and consistent experimental results. The on-fiber hybrid circuits are fabricated with the aid of high-resolution electrohydrodynamic inkjet printing (e-Printing), and the e-Printing achieves feature sizes down to 260 nm on fine fibers in diameters ranging from 1 mm to 60 μm. To the best of our knowledge, this is so far the highest printing resolution on such high-curvature surfaces. The ESOT FiSensor has been validated in diverse scenarios, including complex environmental conditions, aircraft wing condition monitoring, and human-machine interaction.

Compared with conventional optical fiber sensors, the ESOT FiSensor provides several superior advantages. First, it enables simultaneous monitoring and self-decoupling of multiple physical quantities, addressing the longstanding limitations of conventional optical fiber sensors in achieving reliable multimodal decoupling. Second, it is based on a laser-free, self-emissive sensing mechanism, in which on-fiber integrated μLED chips serve as active light sources. This design significantly eliminates bulky external lasers, reducing system volume, weight, and power consumption. Third, the wavelength-multiplexed demodulation strategy enables distributed sensing along extended fiber, the capability of which cannot be realized by either in-fiber functional sensors or near-fiber photodiode detection units. The proposed ESOT FiSensor combines the advantages of electrical and optical sensors, and enables multimodal sensing with long-distance transmission and resistance to EMI. These advantages position the ESOT FiSensor as a compact, integrated, and scalable platform for distributed multimodal perception, and it holds a strong potential for remote human-machine interfaces, aerospace systems, and sensing in electromagnetically harsh environments.

## RESULTS

The signal-sensing process of the ESOT FiSensor is shown in Fig. 1a. External multiple physical signals, such as vibration, temperature, strain, and pressure, are first detected by electrical sensors. These signals are then immediately converted into optical signals by μLEDs in conversion circuits and coupled laterally into the optical fiber, enabling stable long-distance transmission. At the receiving end, a spectrometer demodulates the optical signals based on wavelength modulation, realizing multimodal and distributed sensing. This approach mitigates the attenuation effects associated with electrical wiring in long-distance transmission. It also addresses the key issue of signal distortion in conventional electrical sensors caused by EMI, and it integrates signals from multiple electrical sensors into a single optical fiber, thereby eliminating complex wiring and enhancing spatial efficiency. The multimodal, high-robustness sensing capability endows the ESOT FiSensor with substantial potential for applications in complex environments like geohazard monitoring.

The structural composition of the proposed ESOT FiSensor cell is illustrated in Fig. 1b. The on-fiber circuit consists of *in-situ* printed circuits and flexible printed circuits (FPCs), with discrete components mounted on the surface, including resistors, capacitors, LED chips, and micro-batteries. The encoded optical signals are emitted by LED chips and laterally coupled into the optical fiber for long-distance transmission. Fabrication process primarily relies on high-resolution e-Printing, the technique that can achieve submicron feature sizes that far exceed those of conventional inkjet printing [34,35]. The amplifier and boost converter chips could not be effectively mounted on the fiber because their dimensions exceed the fiber diameter. Therefore, they were integrated onto the FPC instead, which could be compatible with the chip sizes and enable the additional circuit functions. This on-fiber hybrid electronic architecture provides a conformal and scalable platform for fiber-based electronic integration. As shown in Fig. 1c, the e-Printing enables high-resolution pattering on nylon fibers with a diameter of 60 μm, printing microstructures such as serpentine lines, helical coils, and mesh. This compatibility of ultrafine fiber can greatly enhance the flexibility of fiber-based devices. Complex patterns can be directly printed on quartz fibers (*φ* ∼ 250 μm) and polymer optical fibers (POF, *φ* ∼ 1 mm), which include the Chinese characters of ‘Huazhong University of Science and Technology’ and its English abbreviation. The lateral resolution was significantly improved about 40 times to 260 nm on the optical fiber. This implies that the e-Printing holds an exceptional high-resolution manufacturing capability on ultrafine fibers.

To evaluate long-distance signal transmission performance, we compared the strain sensing sensitivity of resistance-based, voltage-based, and ESOT FiSensor (Figs 1d and S1). The electrical sensors and the ESOT FiSensor were tested using a 50 m commercial copper wire (30 AWG) and a 50 m Ultraviolet-Visible (UV-VIS) optical fiber (diameter ∼800 μm), respectively, both for signal transmission. The resistance and voltage signals retained 80.85% and 79.65%, respectively, while the sensitivity retention of the ESOT FiSensor was 90.73% after 50 m of optical fiber transmission. These results demonstrate the superior suitability of the ESOT FiSensor for long-range communication. The immunity to EMI of the proposed sensors was further validated using a low-frequency vibration platform. Under various EMI conditions, the ESOT FiSensor maintained stable signal transmission without obvious distortion withstanding 20 Hz interference. Meanwhile, traditional electrical sensors could not withstand such harsh EMI and exhibited severe signal distortion and instability (Figs 1e and S2). Besides, the ESOT FiSensor holds the distributed sensing capability and the highest functional diversity (Fig. S3), compared with recently reported multimodal fiber sensors [19,26,36–43]. It enables the decoupled detection of four distinct physical parameters, while most existing multimodal sensors are limited to only two or three. Compared with conventional optical fiber sensing schemes, the proposed approach enables scalable multimodal perception and long-distance signal transmission with enhanced robustness against EMI.

**Figure 2.**
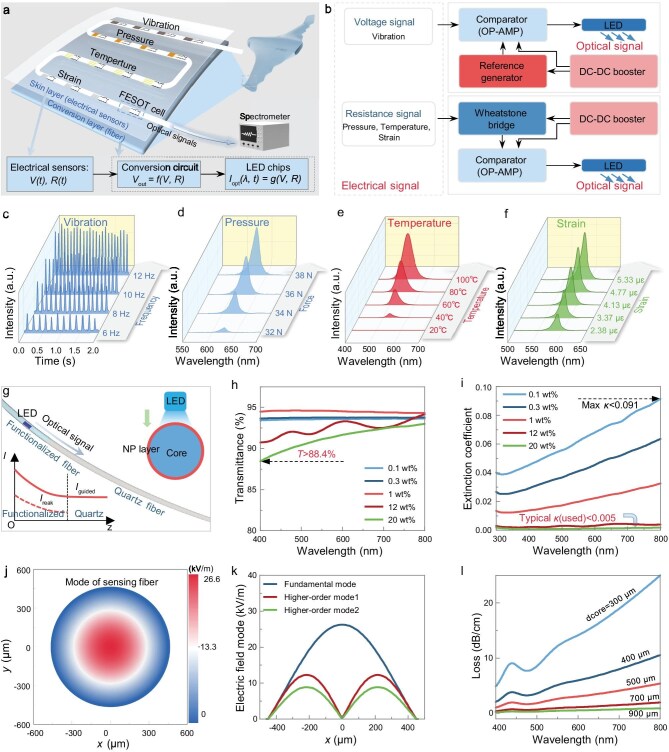
Principles of electrical sensing and optical transmission in the ESOT FiSensor, transmission modes, and loss simulations. (a) Sensing principle and layout of the ESOT FiSensor. (b) Block diagram of two types of conversion circuits based on voltage and resistance inputs. (c–f) Response of the ESOT FiSensor to vibration, pressure, temperature, and strain signals with varying amplitudes. (g) Principle of optical transmission in the ESOT FiSensor. Light from μLEDs is side-coupled into the fiber for transmission. (h) Transmittance of thin nanoparticle films prepared with different nanoparticle concentrations. (i) Extinction coefficient of nanoparticle layers with different thicknesses. (j) Electric field distribution of the typical nc-POF transmission fundamental mode. (k) Electric field distributions of different modes, where the fundamental mode is the primary transmission mode in the fiber. (l) Confinement loss of nc-POF as a function of core diameter.

Figure 2a illustrates the electrical sensing and electro-optical conversion mechanism of the proposed ESOT FiSensor. The resistance (or voltage) signals are directly detected by four types of electrical sensors, and converted (or amplified) into voltage signals by on-fiber conversion circuits. The voltage lights up the on-fiber integrated μLEDs, and the emitted light is laterally coupled into the optical fiber for signal transmission. Multiple ESOT FiSensor cells can be arranged along the fiber to achieve distributed sensing in a programmable manner. Figures 2b and S4 present the block diagram and schematic of voltage-based and resistance-based electro-optical conversion circuits. The two types of conversion circuits share a similar architecture, both assembled with a DC-DC booster and a comparator. The distinction lies in the signal conditioning stage. Voltage-based circuits require a reference voltage for reliable operation, whereas resistance-based circuits employ a Wheatstone bridge to ensure accurate signal conversion. In this configuration, signals from voltage- or resistance-type sensors are translated into the optical intensity of μLEDs. Different sensing channels are distinguished via wavelength modulation, while intensity modulation encodes the quantitative values of each physical parameter. The ESOT FiSensor enables the integration of various electrical sensors at the front end, thereby enabling the detection of multiple physical parameters. As demonstrated in Fig. 2c–f, in multimodal sensing demonstration, this capability allows effective monitoring of low-frequency vibration, pressure, temperature, and strain.

The ESOT FiSensor adopts a dual-section fiber configuration, comprising a front functionalized fiber segment and a rear low-loss quartz fiber segment (Fig. 2g). The functionalized POF segment integrates the electro-optical conversion circuits, while the quartz segment ensures efficient long-distance transmission. To realize efficient side coupling of μLED-emitted light into the fiber, the cladding of the commercial POF was removed via wet etching (Figs S5 and S6). A dielectric Al_2_O_3_ nanoparticle layer was coated on the fiber surface to enhance the stability and resolution of e-Printing [44]. Subsequently, polarization-driven e-Printing was employed to fabricate high-precision interconnect circuits on the nanoparticle-coated POF (nc-POF). To minimize losses during lateral coupling and signal propagation in the sensing segment, the light transmission process was analyzed in detail. According to Lambert-Beer law [45,46], the optical intensity transmitted through the functional fiber segment can be expressed as:


(1) \begin{eqnarray*}
{I}_{{\mathrm{guide}}}(z) = {I}_0\exp ( - 2{\alpha }_s \times z) + {I}_\infty ,
\end{eqnarray*}

where *α*_s_ represents the scattering coefficient of the nanoparticle coating, *z* denotes the position within the optical fiber, *I*_∞_ is the background optical intensity at *z* = ∞, and *I*_0_ is the optical intensity coupled into the fiber from the μLED source. These results indicate that transmission loss decreases as the scattering coefficient of the nanoparticle film is reduced. The lateral coupling mechanism can be considered as the superposition of two contributions. One is the intrinsic coupling determined by the angular divergence of the light source. The other is the enhanced coupling induced by nanoparticle scattering. Theoretical analysis shows that an increase in the scattering coefficient (*α*_s_) strengthens local lateral coupling but simultaneously exacerbates propagation losses within the fiber. Hence, the optimal coupling efficiency is governed by a trade-off between scattering-induced enhancement and transmission loss (Fig. S7). Since intrinsic coupling dominates the coupled optical intensity, reducing the scattering coefficient of the nanoparticle layer is beneficial, which can be achieved by preparing more uniform films using nanoparticles with much smaller size.

To evaluate its potential influence on optical transmission, nanoparticle films in different thicknesses were prepared by dip-coating of aluminum oxide nanoparticles dispersions with different mass fractions. The transmittance, reflectance, and absorbance of these films were characterized by a UV-Vis spectrophotometer (Fig. S8), and the transmittance generally exceeds 88.4% in the visible range (400–800 nm) (Fig. 2h). Notably, the transmittance surpasses 92.5% with a film in thickness below 500 nm, and the reflectance and absorbance remain below 8%. This implies that the nanoparticle layer is nearly transparent in the visible spectrum and enables efficient emitted light coupling from as-mounted μLEDs into the optical fiber core. To further characterize the photon absorption capability of nanofilm, the absorption coefficient *α* was calculated and analyzed as follows [47]:


(2) \begin{eqnarray*}
\alpha = - \frac{1}{d}\ln \left[ {\frac{{{{(1 - R)}}^2}}{{2T}} + \sqrt {{R}^2 + \frac{{{{(1 - R)}}^4}}{{4{T}^2}}} } \right],
\end{eqnarray*}

where *d, T*, and *R* are the thickness, transmittance and reflectance of the film, respectively. Both *T* and *R* are functions of the wavelength *λ*. Additionally, the extinction coefficient *κ* of the material can be derived from the absorption coefficient:


(3) \begin{eqnarray*}
\kappa = \frac{{\alpha \lambda }}{{4\pi }}.
\end{eqnarray*}

As the film thickness increases from the submicron to the micrometer scale, the absorption coefficient *α* and the extinction coefficient *κ* in the visible range decreases gradually (Figs 2i and S9a). This is primarily attributed to structural changes in the film. Thin films with thickness in submicron scale tend to be less dense and contain more voids and surface defects, which act as scattering centers and lead to stronger light scattering. However, there are fewer interparticle gaps and defects in compact films with thickness around 1 μm. This reduces scattering losses and results in a lower apparent absorption coefficient. A similar trend is found for the extinction coefficient *κ*, which includes both absorption and scattering contributions and reflects the overall optical attenuation of the film. In the Tauc plot used to characterize the absorption coefficient as a function of photon energy (Fig. S9b), the linear portion is extended to (*αhv*)² = 0. This extension enables the determination of the bandgap *E_g_* for films with different thicknesses (Fig. S9c). The bandgap of the nanoparticle films generally ranges from 4.2 eV to 4.8 eV, which is smaller than the bandgap of bulk aluminum oxide (8.7 eV). This reduction is attributed to the higher defect density in the nanoparticle films compared to bulk materials [48]. The increased compactness in thicker films enhances potential quantum contact effects, leading to a lower bandgap. This is consistent with previously reported changes in film resistivity [44]. The refractive index of the aluminum oxide nanoparticle film gradually decreases with increasing wavelength as shown in Fig. S9d. Although the refractive index values vary with the film thickness, the trend and values are generally consistent with the theoretical refractive index [49,50]. This agreement provides indirect confirmation of the accuracy of the preceding theoretical and experimental results.

To analyze the electric field distribution and optical loss of the nc-POF, a corresponding simulation model was established using COMSOL (Fig. S10). In this model, *d*_1_ and *n*_1_ represent the diameter and refractive index of the fiber core [e.g. poly(methyl methacrylate)]. The *d*_2_ and *n*_2_ correspond to the diameter and refractive index of the cladding (e.g. aluminum oxide). A perfectly matched layer was added outside the cladding to simulate optical absorption in an infinitely extended environment. The typical fundamental mode distribution in the nc-POF is shown in Fig. 2j. Compared to higher-order modes (Figs 2k and S11a–c), the fundamental mode exhibits strong electric field confinement within the core region and gradually decays outward (Fig. S11d–e). It is the primary mechanism for long-distance transmission in the fiber. The effective refractive index *n*_eff_ remains approximately equal to the core refractive index *n*_core_ across different wavelengths (Fig. S12a). This is a characteristic feature of the fundamental mode. Figure S12b presents the dispersion curves of the core and cladding from previous work [49,50], illustrating the wavelength-dependent variation in their refractive indices. Figure S13a–b present the variation of the radial electric field distribution in the nc-POF as a function of operating wavelength. It can be observed that the nc-POF exhibits higher electric field intensity at shorter wavelengths. The *CL* is used to characterize the light-guiding efficiency of the nc-POF and is defined as:


(4) \begin{eqnarray*}
CL = 8.868 \times {k}_0 \times {I}_m[{n}_{{\mathrm{eff}}}] \times {10}^4,
\end{eqnarray*}

where *k*_0_ is the free-space propagation constant, given by *k*_0_ = 2 π/*λ. I*_m_[*n*_eff_] represents the imaginary part of the effective refractive index *n*_eff_, and *λ* is the operating wavelength. Therefore, the CL is primarily influenced by the imaginary part of the refractive index, which allows for its quantitative estimation. Simulation results indicate that as the core diameter increases, the peak electric field intensity gradually rises, possibly due to stronger optical confinement (Fig. S13c–d). Figure 2l shows that the CL decreases as the core diameter increases. For instance, as the core diameter increases from 300 μm to 900 μm, the CL decreases from 5.41 dB/cm to 0.2 dB/cm at 405 nm accordingly (Fig. S14). Based on these insights, a fiber core with a relatively large-diameter is preferred for the fabrication of nc-POF, i.e., by removing the cladding of the original POF and dip-coating with a nanoparticle layer to facilitate e-Printing. To evaluate the optical loss in the functionalized fiber, the cutback method was applied to three types of POFs (Fig. S15): original POF, air-cladded POF, and nc-POF. Under 405 nm laser transmission, the measured loss of the nc-POF was 0.081 dB/cm, which is close to the simulated value in Fig. 2l. The slight deviation can be attributed to scattering-assisted light confinement that is induced by the nanoparticle layer, which enhances mode coupling and partially compensates for intrinsic transmission losses. The nc-POF showed slightly higher loss than the original POF. Fortunately, the loss remains well within acceptable limits for long-distance transmission, because the electro-optical conversion occurs only in a short functional segment at the fiber front end.

**Figure 3.**
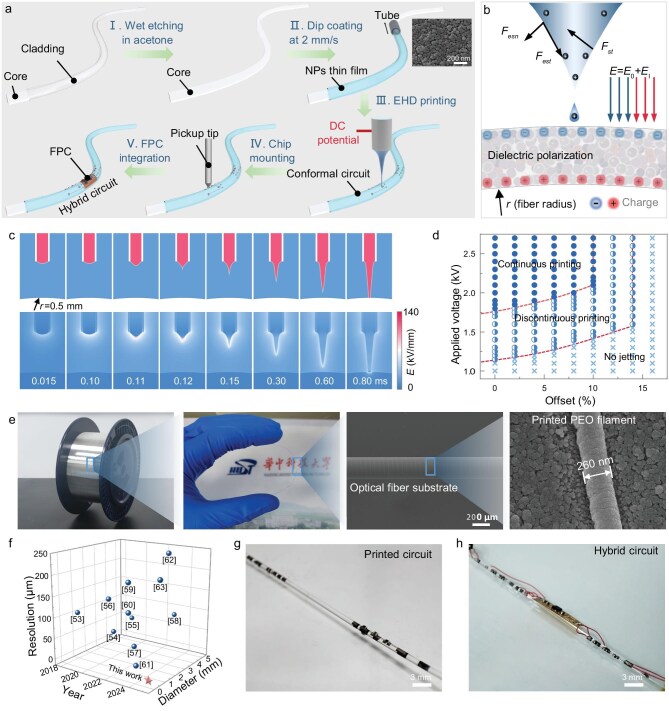
Principle and control of functional structure fabrication on the surface of optical fiber. (a) Fabrication process of the hybrid interconnect circuit on the fiber surface. (b) Mechanism of polarization-driven e-Printing on the fiber surface. (c) Simulation of the e-Printing process on a micro-cylindrical substrate with a diameter of 1 mm, showing fluid volume fraction and electric field distribution during jetting. (d) Map of the relationship between the nozzle offset distance and the continuous printing state on a fiber substrate with a diameter of 1 mm. (e) Micro-nanostructures printed on the optical fiber (*φ*∼250 μm), with a maximum resolution of 260 nm. (f) Comparison of e-Printing with other micro-cylindrical printing techniques in terms of compatible diameters and fabrication resolution. (g) Fiber-surface printed circuit after chip integration. (h) A hybrid circuit integrated with FPC.

Figure 3a illustrates the fabrication process of the on-fiber electro-optical conversion circuits. The cladding of a commercial POF (*φ* ∼ 1 mm), for example, was first partially stripped by wet etching to expose its core (*φ* ∼ 900 μm). Then a dielectric nanoparticle layer was deposited onto the fiber core by dip-coating. This layer would induce field-driven polarization, regulating local charge distribution that can stabilize the e-Printing process (Fig. 3b). Conformal circuits were directly printed onto the fiber surface subsequently. Discrete components such as resistors, capacitors, and LEDs were integrated and interconnected with an FPC that sets functional chips. The resulting architecture forms a conformal hybrid circuit on the fiber surface (Fig. S16). This mask-free inkjet printing could provide submicron precision and compatibility with substrates of diverse configurations [34,51,52]. Figure 3b schematically depicts the principle of e-Printing on ultrafine fiber. A positive DC voltage was applied to the nozzle to generate a strong localized electric field, leading to the formation of a Taylor cone with a diameter one to two orders of magnitude smaller than the nozzle orifice, where the dielectric nanoparticle layer plays a critical role [44]. Through Maxwell-Wagner polarization, it establishes localized surface charges that neutralize the residual charge from deposited droplets, thereby preventing mutual repulsion. The induced polarization field further superimposes on the applied electric field, significantly reducing the required onset voltage and mitigating breakdown risks under high voltages. Unlike conventional e-Printing, this polarization-driven method does not require a grounded electrode, enabling high-resolution printing on ultrafine cylindrical substrates. Moreover, the nanoparticle layer exhibited minimal impact on the interfacial adhesion between as-printed structures and the substrate as shown in Fig. S18. This ensures reliable integration without compromising mechanical stability.

The e-Printing process on a fiber substrate (e.g. a radius of 500 μm) was validated through finite element simulations (Figs 3c and S19). Silver ink and a quartz glass nozzle were employed. Under the applied electric field, the meniscus at the nozzle tip gradually elongated to form a Taylor cone, which subsequently deposited onto the fiber substrate. At 2500 V, the formation and deposition of the Taylor cone took approximately 0.6 ms. Notably, the electric field is primarily concentrated at the nozzle during the e-Printing process, and the electric field strength at the substrate surface remains relatively weak. Hence, the electric field configuration would not induce aggregation of dielectric nanoparticles on the substrate, nor does it adversely affect the uniformity of deposited ink. Deviations in substrate coaxiality or nozzle positioning could affect the printing on high-curvature surfaces. For a 500 μm-radius substrate, continuous printing became challenging when the lateral offset exceeded 50 μm (Fig. 3d). The effects of printing height and applied voltage on the printing behavior were systematically investigated. Generally, a higher applied voltage would enhance ink ejection, leaving wider printed lines and lower line resistance (Fig. S20a–b). These results offer valuable guidance for process optimization to enhance resolution. The method allows fine patterning on fiber in diameters ranging from 60 μm to 5 mm, and can achieve submicron structures with a lateral resolution of 260 nm on a 250 μm-diameter quartz fiber (Fig. 3e). It is also compatible with diverse ink materials such as silver paste and perovskites (Fig. S20c–d). Compared with existing micro-cylindrical manufacturing techniques [53–63], the e-Printing can be compatible with the smallest cylinder diameters and achieve the highest fabrication resolution reported to date (Fig. 3f). A representative printed circuit was directly fabricated on nc-POF by e-Printing, along with the hybrid electro-optical circuits integrated with FPC (Fig. 3g–h). These results demonstrate the capability of the proposed e-Printing approach for precise and reliable microfabrication.

**Figure 4.**
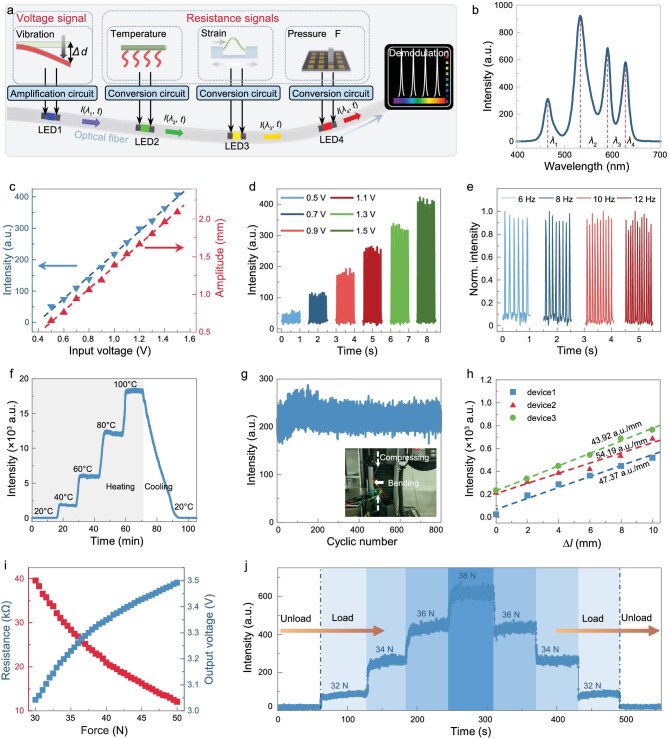
Multiparameter sensing validation based on the ESOT FiSensor. (a) Schematic illustration of the ESOT sensing process for multiple physical stimulations including vibration, pressure, temperature, and strain. (b) Typical full spectra of the four wavelength-modulated signals. (c) Relationship between the exciter’s amplitude and the transmitted optical intensity as a function of the input voltage. A piezoelectric sensor is used at the front end. (d) Response of the ESOT FiSensor to 10 Hz vibration signals with different amplitudes. (e) Response curves of the ESOT FiSensor to vibration signals at different frequencies at an input voltage of 2 V. (f) Response of the ESOT FiSensor to a stepwise temperature signal, using a thermistor as the front-end electrical sensor. (g) Variation of the transmitted optical intensity of the ESOT FiSensor during 800 bending cycles at room temperature. (h) Sensitivity curves of different ESOT FiSensor devices for strain sensing. (i) Variation in resistance of the piezoresistive sensor and the output voltage of the conversion circuit under different pressures. (j) Optical intensity response of the ESOT FiSensor under stepwise pressure loading.

The proposed ESOT FiSensor enables the integration of four electrical sensors, supporting multiple physical stimulation monitoring in a single optical fiber (Fig. 4a). It converts electrical signals, including voltage and resistance, into optical signals and supports their long-distance transmission and demodulation. Vibrations, temperature, strain, and pressure were taken as functional examples. Signals from different sensor types were distinguished using wavelength-division multiplexing. The optical intensities at 467 nm, 536.6 nm, 593.4 nm, and 631.8 nm were monitored to extract the relative values of each physical quantity (Fig. 4b).

For vibration monitoring, an external commercial piezoelectric sensor was adopted to achieve electro-optical sensing of low-frequency vibration signals (Fig. S21a). The voltage generated by the piezoelectric sensor lights up the μLED. Vibration signals with different amplitudes and frequencies were detected at the receiving end by means of optical intensity and frequency monitoring. The exciter amplitude was controlled by varying the input voltage generated by a dynamic signal analyzer (DSA). Variations in the transmitted spectrum at a fixed vibration frequency of 10 Hz under different input voltages are shown in Fig. S21b. Both the exciter amplitude and transmitted optical intensity exhibit a linear correlation with the voltage (Fig. 4c). This indicates a strong linear relationship between the optical intensity and the amplitude. The measured sensitivity is 245.7 a.u./mm, with a detectable amplitude range of 0.6 mm to 2.1 mm (Fig. S21c). By extracting the optical intensity at 467 nm under different input voltages, a distinct step variation from 0.5 V to 1.5 V can be measured (Fig. 4d). Normalizing the transmitted optical intensity allows the ESOT FiSensor to detect different vibration frequencies (Fig. 4e). Higher-frequency signals required a spectrometer with a higher sampling rate. At a vibration frequency of 20 Hz, the sensitivity of the ESOT FiSensor exhibits slight variations (Fig. S21d–f). This is due to the simultaneous change in exciter amplitude when adjusting the frequency signal via the DSA (Fig. S21g).

By adopting a PT100 temperature-sensitive resistor, the ESOT FiSensor is capable of monitoring temperature stimuli. The sensor can accurately capture and respond to stepwise temperature variations below 100 °C, both heating and cooling processes (Fig. 4f). During 10 cyclic thermal detecting between 35°C and 50°C, the sensor still exhibited excellent stability and repeatability (Fig. S22a). It also can detect temperature differences on the sidewalls of water cups at room temperature, 50°C, and 80°C, as well as temperature fluctuations induced by fingertip contact and release (Fig. S22b and c). Besides, cyclic bending tests were conducted at room temperature to evaluate the mechanical durability of the ESOT FiSensor (Fig. 4g). The emission intensity remained stable during 800 cyclic bending, and its sensitivity still kept highly linear after the bending cycles (Fig. S22d). The device can bear bending and torsion to some extent as well (Fig. S22e and f), confirming the robustness of the ESOT FiSensor module under mechanical deformation. The electro-optical strain sensing was validated with a flexible strain sensor (Fig. S23a). It shows a distinct response to gradient variations in compression displacement (Fig. S23b), presenting a relatively excellent sensing stability upon multiple cycles (Fig. S23c and d). The intensity degradation remains within 5% after 600 cyclic compressions at 40 mm displacement, presenting its robustness for long-term strain monitoring. To evaluate device-to-device reproducibility, multiple independent ESOT FiSensor devices were characterized for strain sensing (Fig. 4h). The response curves of three devices exhibit high linearity with very similar slopes. The extracted sensitivities are closely matched among such devices, showing their nice reproducibility.

By adopting a flexible pressure sensor, ESOT FiSensor can detect pressure signal, which is difficult to realize by conventional optical fiber sensors (Fig. S24). The variations in sensor resistance and the corresponding output voltage from the voltage conversion circuit under different pressure levels are illustrated in Fig. 4i. The sensor can resolve changes as small as 0.5 N and exhibits a clear response to stepwise pressure inputs (Figs 4j and S25a). Repeated finger touches, either at constant or varying pressure levels, can be reliably detected (Fig. S25b and c). The electro-optical response time is less than 15 ms (Fig. S25d), and the stability was also validated by over 100 repeated presses (Fig. S25e). Except for commercial sensing units adopted in the initial demonstrations, several other electrical sensing units including pressure, temperature, and strain sensors were also fabricated via e-Printing (Fig. S27). Their electrical outputs are compatible with electro-optical conversion circuits through appropriate parameter matching. These results provide a pathway toward a fully printed ESOT FiSensor system.

**Figure 5.**
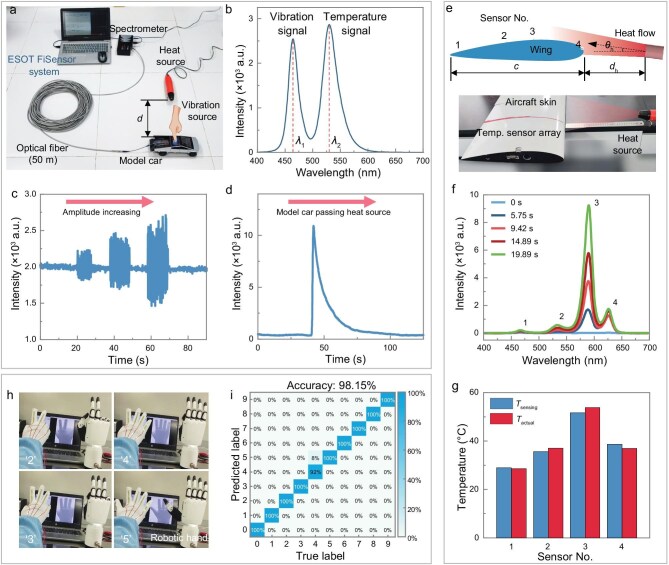
Functional demonstrations of the ESOT FiSensor in different application scenarios. (a) Synchronous monitoring of vibration and temperature signals in a moving model car. (b) Typical spectrum showing wavelength-modulated vibration and temperature signals. (c) Detected signal intensity of the model car increases with external excitation amplitude. (d) Signal variations detected when the model car passes by a heat source. (e) Distributed temperature monitoring on an aircraft wing surface using an integrated temperature sensor array. (f) ESOT FiSensor responses at four temperature points under continuous heat flux, in which each point is wavelength-modulated. (g) Comparison between detected and actual temperatures after 30 s of heating at a distance of 30 cm. (h) Real-scene demonstration of real-time gesture recognition and robotic hand control enabled by the ESOT FiSensor based on a strain gauge array. (i) Trained confusion matrix for gesture recognition based on the ESOT FiSensor.

To validate the multimodal sensing capability of the ESOT FiSensor under complex environmental conditions, an experiment was conducted on a model car subjected to coupled vibration and temperature stimuli (Fig. 5a). Vibration and temperature sensors were mounted on the chassis and sidewall of the model car, respectively. The collected signals were converted into optical signals and transmitted through a 50 m long optical fiber. Wavelength multiplexing of different μLEDs enabled multimodal signal encoding, allowing independent detection of vibration and temperature inputs (Fig. 5b). When different vibration amplitudes were applied to the car by a finger, variations in intensity of transmitted light were effectively detected at the receiving end (Fig. 5c). The optical response was also monitored as the car passed over a localized heat source simulated by a heat gun (Fig. 5d). The surface temperature rose sharply and then gradually decreased during the whole process. As the car remained stationary (Fig. S28), the measured temperature decreased with increasing distance from the heat source. This trend agrees well with the thermocouple measurements, with a maximum deviation of 4.8%, showing its high capability to spatially resolve environmental thermal variations.

The ESOT FiSensor was also mounted on wing surfaces of an aircraft to extend the distributed sensing capability of intelligent flexible sensing skin [64,65]. Four temperature sensors were integrated onto a wing model as shown in Fig. 5e. The signals were transmitted through a 50 m optical fiber to capture the spatial temperature distribution across the wing surface. The sensor arrays were positioned at 0.02*c*, 0.4*c*, 0.6*c*, 0.95*c*, where c denotes the chord length (Fig. S29). Figure 5f presents the full optical spectrum with a thermal source placed 20 cm from the wing surface that experiences continuous heating. The optical intensity at different wavelengths was analyzed and mapped to calibration curves. The detected temperature at each sensing node during heating was obtained (Fig. S30a–h). The temperature at all positions decreased as the heating source was set at 30 cm accordingly (Figs S30i–l and S31a). A comparison between detected temperatures after heating for 30 s with reference thermocouple measurements showed errors within 4.3% (Figs 5g and S31b). The ESOT FiSensor enables multiplexed transmission of signals from multiple sensors through a single optical fiber. The channel capacity is only limited by the μLED full width at half maximum (FWHM) and the available spectral bandwidth. Herein, four sensing channels are adopted to demonstrate distributed monitoring. In future extensions, the number of resolvable channels can be further increased by employing μLED sources with much narrower FWHM. This approach greatly reduces interconnecting wire requirements in constrained spaces and extends the transmission distance of aircraft skin signals, underscoring its potential for next-generation applications.

Gesture recognition and remote human-machine interaction represent key application areas for flexible optoelectronic technologies [66,67]. As shown in Fig. 5h, a strain-based ESOT FiSensor array was employed to enable both gesture recognition and interaction control. The LEDs array on the fiber surface exhibits distinct emission spectra under different gestures. An artificial neural network can be trained by analyzing spectral information to achieve human-machine interaction. Hand gesture information was captured by the ESOT FiSensor. The data were then processed using a t-distributed stochastic neighbor embedding algorithm for dimensionality reduction (Figs S32–S37). By optimizing the architecture of an artificial neural network, accurate classification of ten distinct gestures was achieved, yielding a recognition accuracy of 98.15% (Fig. 5i). Based on the classification results, robotic manipulators were controlled in real time to perform the corresponding gesture actions with high precision. This reveals the potential of the ESOT FiSensor for human-machine interaction applications.

## CONCLUSION

In summary, we present an ESOT FiSensor based on on-fiber hybrid electro-optical circuits. This FiSensor enables simultaneous and distributed detection of four physical stimuli—vibration, pressure, temperature, and strain—while maintaining long-distance, interference-resistant optical transmission. Systematic experiments demonstrate its reliable multimodal monitoring with optical signal demodulation and low inter-channel crosstalk across distributed fiber channels. The e-Printing holds a printing resolution of 260 nm on fine fibers in diameters down to 60 μm, and it allows direct, conformal fabrication of microcircuits on optical fibers to establish ESOT FiSensor. The sensor was further validated across diverse scenarios, including robust multimodal sensing under simulated complex conditions. It permits both spatial temperature mapping on aircraft wings and gesture recognition for human-machine interaction. Endowing optical fibers with multimodal perception capabilities addresses key limitations of conventional fiber sensing and opens up broad application prospects. Besides, the ultrafine fiber endows the device with exceptional flexibility, facilitating its integration into soft and conformable applications. Future developments could realize a fully printed ESOT FiSensor on the optical fiber, incorporating both functional chips and circuits through conformal printing to further enhance device integration. Conceptually, it offers a pathway to transform billions of kilometers of optical fiber from passive information carriers into active, distributed, multimodal sensing platforms. Such a transformation holds substantial promise for broad deployment in emerging applications, including fiber-enabled unmanned aerial vehicles, electric vehicles, and subsea earthquake monitoring systems.

## MATERIALS AND METHODS

### Materials

Polymer optical fibers (CK-40) were purchased from Mitsubishi Chemical Co., Ltd. UV-VIS quartz optical fibers (∼800 μm diameter) for long-distance transmission were supplied by Guangzhou Ruike Photoelectric Technology Co., Ltd. The copper wire (30 AWG) was purchased from Shenzhen RSW Technology Co., Ltd. Acetone was obtained from Sinopharm Chemical Reagent Co., Ltd. Silver ink (100 cp, 75%) was sourced from Beijing BroadTeko Technology Co., Ltd. Aluminum oxide nanoparticle dispersion was acquired from Hangzhou ZhiTai Purification Technology Co., Ltd. Piezoelectric ceramic sheets were obtained from Zhejiang Shenlei Ultrasonic Technology Co., Ltd. Flexible pressure sensors were purchased from Luoyang GuanTuo Electronics Technology Co., Ltd. Strain sensors were sourced from Shenzhen RunesKee Technology Co., Ltd. Temperature sensors (PT100) were purchased from Heraeus Group.

### Fabrication of the ESOT FiSensor

A commercial POF (*φ*∼1 mm) was stripped at the front end to expose a region for lateral coupling. The exposed segment was immersed in acetone for 15 min to remove the cladding, yielding a ∼900 μm core. A thin film of aluminum oxide nanoparticles was then deposited onto the core surface via dip-coating and allowed to dry naturally. The fiber was subsequently fixed on a micro-fiber clamping device. Interconnect circuits were fabricated on the fiber surface by depositing silver ink via an e-Printing platform. A quartz glass nozzle with a diameter of 10 μm was employed, with a nozzle-to-substrate distance of 20 μm, an applied voltage of 2 kV, and a substrate speed of 0.5 mm/s. After printing, the deposited silver patterns were annealed at 70°C for 2 h to form conductive interconnect circuits. Discrete components, including resistors, capacitors, and LEDs, were precisely mounted using a micromanipulation platform. Electrical interconnection with the FPC was achieved using soldering at the FPC terminals and high-viscosity conductive silver paste at the printed circuit terminals. The two sides were electrically connected via wires, forming a hybrid circuit. By electrically integrating the hybrid circuit with front-end sensors, the ESOT FiSensor was constructed.

## Supplementary Material

nwag250_Supplemental_File
