# Supplementary material for: Flexible, multimodal, electrical-sensing–optical-transmission μfiber-sensors via an on-fiber printed electronics strategy
Source: Natl Sci Rev. 2026 May 7;13(12):nwag250. doi: 10.1093/nsr/nwag250 (PMC13296559; doi:10.1093/nsr/nwag250)
Supplement: nwag250_Supplemental_File [file nwag250_supplemental_file.pdf]

Supplementary Information

Flexible, Multimodal, Electrical-Sensing-and-Optical-Transmission μFiber-Sensors via an on-Fiber Printed Electronics Strategy

Hongyang Wang[a,b,c], Dong Ye[a,b,c,*], Qingshuang Wu[a,b,c], Longkang Yang[a,b,c], Chenyang Zhao[a,b,c], Dada Wu[a,b,c], Yongqi Guo[a,b,c],Wangyang Xu[d], Wei He[a,b,c], Zhangyu Xu[a,b,c], Hao Wu[a,b,c], Wenlong Lu[a], Wei Li[e], Qizhen Sun[d], and YongAn Huang[a,b,c,*]

[a] State Key Laboratory of Intelligent Manufacturing Equipment and Technology, Huazhong University of Science and Technology, Wuhan 430074, P.R. China

[b] Research Center for Advanced Electronics Manufacturing, Huazhong University of Science and Technology, Wuhan 430074, P.R. China

[c] Flexible Electronics Research Center, Huazhong University of Science and Technology, Wuhan 430074, P.R. China

[d] School of Optical and Electronics Information, National Engineering Research Center of Next Generation Internet Access System, Huazhong University of Science and Technology, Wuhan, Hubei, 430074, P.R. China

[e] AVIC The First Aircraft Institute, Xi'an, Shaanxi, 710089, P.R. China

*To whom correspondence should be addressed. E-mail: yahuang@hust.edu.cn, yedong@hust.edu.cn.

1. Experimental Section

Electromagnetic interference resistance testing

A low-frequency vibration platform (Y-DC-10C, Wuxi Yibaofan Environmental Test Equipment Co., Ltd.) was used to apply electromagnetic interference to the sensor. During operation, the vibration platform generated electromagnetic interference signals corresponding to its vibration frequency. To evaluate the EMI resistance of the sensors, a 1 m wire of electrical strain sensor and a 1 m optical fiber of the ESOT FiSensor were separately placed on the vibration platform for testing. The resistance variation of the electrical sensor and the optical signal intensity change of the ESOT FiSensor were measured to assess their respective resistance to electromagnetic interference.

Multimodal signal monitoring based on the ESOT FiSensor

Vibration signal detection: A dynamic signal analyzer (Spider-20E, Crystal Instruments) generates excitation signals, which are amplified by a power amplifier and used to drive a shaker (Modal 110 Exciter System, MB Dynamics) at controlled frequencies and amplitudes. The actual frequency and amplitude are simultaneously captured by the DSA and analyzed on a host computer. A piezoelectric sensor (PZT-based) converts the vibration of the shaker into electrical signals, which in turn modulate the emission intensity of the LED on the side of the nc-POF. Changes in vibration amplitude or frequency lead to corresponding variations in LED intensity and emission frequency. The transmitted optical spectrum is detected using a spectrometer (Maya2000, OceanView Installation), enabling real-time monitoring of external vibration signals.

Pressure signal detection: Specific pressure values are applied using a pressure tester (DS2-50N, ZHIQU), where a rectangular pressing block transfers the force onto a 0.64 mm^2 pressure sensor. The resulting resistance variation of the sensor is converted into voltage changes through a voltage conversion circuit, subsequently modulating the LED intensity on the lateral surface of the nc-POF. The transmitted optical spectrum is then detected by a spectrometer, enabling electro-optical sensing of pressure signals. Additionally, non-specific pressure values are introduced via finger pressing, following the same testing procedure.

Temperature signal detection: A constant temperature and humidity test chamber (JY-S, Shenzhen Changxu Mechanical Equipment Co., Ltd.) was used to provide a stable thermal environment. A front-end temperature sensor (PT100) detected temperature variations, which were converted into optical intensity changes on the surface of the nc-POF via a signal conversion circuit. These optical signals were transmitted through the optical fiber and subsequently demodulated by a spectrometer to retrieve the temperature information.

Strain signal detection: A pneumatic grip of a compression tester (Instron 5944) holds the strain sensor (sensor region: 6 mm; gripping length: 10 mm). The strain sensor undergoes controlled compression displacements using the testing machine, leading to resistance variations that are converted into voltage changes via a voltage conversion circuit. This modulates the LED intensity on the nc-POF surface. The transmitted optical spectrum is then demodulated by a spectrometer, enabling precise strain measurement.

Multimodal sensing under simulated natural disasters

To demonstrate the sensor's capability for multimodal monitoring in simulated disaster scenarios, a flexible PVDF piezoelectric sensor and a PT100 temperature sensor were mounted on the chassis and sidewall of a mobile platform, respectively, serving as front-end electrical sensing units. The outputs from these sensors were amplified and converted into driving voltages for LEDs integrated on the fiber surface, generating modulated optical signals. Manual pressing with varying force was used to simulate different levels of mechanical vibration, while a hot air gun was applied to mimic a localized high-temperature source. Wavelength division multiplexing was employed to enable simultaneous monitoring of both vibration and temperature signals. The optical signals were transmitted through a 50 m optical fiber and subsequently demodulated by a spectrometer to extract the respective signal intensities.

Distributed temperature monitoring on the wing surface

Four PT100 sensors were mounted at 0.02*c*, 0.4*c*, 0.6*c*, and 0.95*c* along the wing chord (*c* = 35.8 cm, where *c* denotes chord length). To minimize the system footprint on the fiber surface, part of the conversion circuit was implemented on a printed circuit board independent of the fiber, while the fiber-mounted LED array was connected to the circuit through printed interconnects. The LEDs emitted at 467 nm, 536.6 nm, 593.4 nm, and 631.8 nm, enabling wavelength-based distinction of sensing points. A heat gun positioned 20 or 30 cm from the leading edge and angled at 8° delivered airflow along the chord for 30 s. Real-time spectral changes were recorded and converted to temperature values using calibration curves, with simultaneous thermocouple measurements serving as references.

Gesture recognition and human-machine interaction

Smart glove configuration: Strain sensors were attached to the PIP joints of all five fingers of the glove using high-adhesion E7000 textile glue, ensuring seamless integration with finger movements. The sensor signals were routed via conductive wires and secured onto the glove using high-adhesion 3M tape.

Spectral data acquisition: The arrayed strain sensors were interfaced with a conversion circuit to drive the corresponding LED array. The optical signals emitted by the LEDs were

coupled into the fiber and transmitted to a spectrometer at the receiving end. A custom C-based software platform was developed to acquire and display real-time optical signal variations. Timer parameters were adjusted to enable real-time data storage, and thread locks were implemented to maintain data consistency.

Gesture recognition: A hybrid A convolutional neural network–long short-term memory (CNN-LSTM) architecture was developed for spatiotemporal feature extraction, with t-SNE applied for dimensionality reduction. Optical intensity patterns corresponding to distinct gestures were normalized and partitioned into training/test datasets (7:3 ratio). Spatial features were extracted via four 1D convolutional layers, while temporal dependencies were captured by LSTM networks. The output layer classified gestures based on different optical signals.

Human-machine interaction control: The robotic hand (Inspire-Robots) was controlled via a serial communication protocol. Real-time gesture data were formatted into feature vectors and fed to the trained model for instantaneous classification. Recognized gestures were displayed on-screen simultaneously triggering the robotic hand to perform the corresponding movements with precise synchronization.

Channel capacity and crosstalk suppression of the ESOT FiSensor

The channel capacity of the ESOT FiSensor is intrinsically constrained by the full width at half maximum (FWHM) of the μLEDs and the available optical bandwidth W. To maintain the robustness of the decoupling algorithm, the theoretical maximum number of channels (N) can be estimated as:

$$N \approx \frac{W}{k \cdot \Delta\lambda_{FWHM}} \tag{S1}$$

Here, $\Delta\lambda_{FWHM}$ represents the average FWHM of the μLED emission spectra. The parameter k is an interval factor introduced to reduce computational error and improve channel separation stability. In this study, the μLEDs used have an average FWHM of 22.36 nm. To reduce spectral crosstalk, k=3 is adopted. Within the visible spectral range (400~800 nm), the theoretical maximum number of resolvable channels is therefore N≈6. In this study, considering that the peak wavelengths are not uniformly distributed across the spectrum, we demonstrate distributed monitoring using four sensing channels. In future developments, further increases in channel capacity could be achieved by reducing the spectral linewidth of the μLEDs through innovations in LED materials and device structures. For example, if the FWHM of the μLED emission spectra could be reduced by half, the theoretical number of resolvable channels would approximately double, reaching about 12 channels.

Considering that intensity variations in one sensing channel may induce cross talk in adjacent channels, a pre-calibrated decoupling matrix can be applied to ensure reliable self-decoupling. Even in the presence of slight spectral overlap, the original signals can be accurately reconstructed by solving the corresponding matrix equation. The decoupling model is expressed as:

$$\begin{pmatrix} I(\lambda_1) \\ I(\lambda_2) \\ \vdots \\ I(\lambda_n) \end{pmatrix} = \begin{pmatrix} K_{11} & K_{12} & \cdots & K_{1n} \\ K_{21} & K_{22} & \cdots & K_{2n} \\ \vdots & \vdots & \ddots & \vdots \\ K_{n1} & K_{n2} & \cdots & K_{nn} \end{pmatrix} \begin{pmatrix} S_1 \\ S_2 \\ \vdots \\ S_n \end{pmatrix} \tag{S2}$$

Here, $I(\lambda_i)$ denote the total detected intensity at the i-th wavelength channel. S_i represents the true physical signal of the corresponding sensing unit. K_{ij} is the pre-calibrated cross-sensitivity coefficient. The diagonal terms (i=j) correspond to the primary response, while the off-diagonal terms (i≠j) represent cross-talk contributions. The original signals can be obtained through matrix inversion, S=$K^{-1}I$, which effectively suppresses inter-channel interference and signal distortion.

2. Supplementary Figures

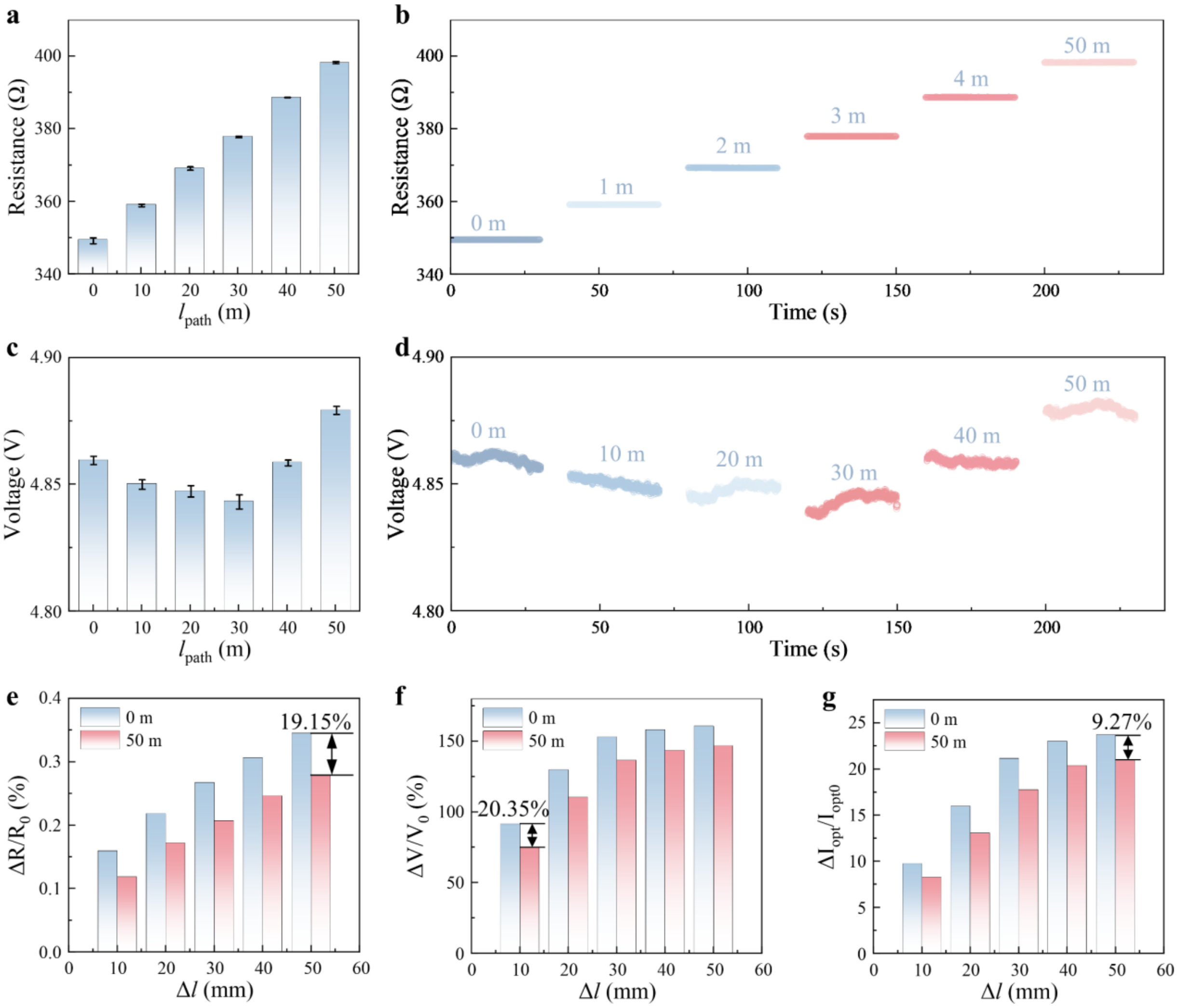

Figure S1. Long-distance transmission performance of electro-optical and purely electrical sensors. (a), (b) Detected resistance signals and corresponding mean values at different transmission distances. (c), (d) Detected voltage signals and corresponding mean values at different transmission distances. (e–g) Sensitivity degradation at a transmission distance of 50 m for resistance-based electrical sensors (e), voltage-based electrical sensors (f), and the ESOT FiSensor (g).

To systematically evaluate the long-distance transmission performance of the ESOT FiSensor, comparative tests were conducted with resistance-based, voltage-based sensors. These sensors were tested over 50 m of commercial copper electrical wire (30 AWG) commonly used in device measurements, while the ESOT FiSensor was tested over 50 m of quartz optical fiber (UV–VIS fiber, diameter ~800 μm). The resistive signal was obtained by directly measuring the resistance change of the flexible strain sensor using a digital multimeter. The voltage signal was measured after converting the resistance variation into a voltage output through a signal conversion circuit. As shown in **Figure S1a-d**, with increasing transmission distance, the resistive signal exhibited a gradual increase, while the voltage signal showed

noticeable fluctuations. This behavior arises from the high susceptibility of voltage transmission to external electromagnetic interference. **Figure S1e-g** compare the strain sensitivity retention after 50 m transmission. Resistance-based, voltage-based sensors retained approximately 80.85% and 79.65% of their original sensitivity, respectively, showing significant attenuation over distance. In contrast, the ESOT FiSensor converts the front-end electrical signal into an optical signal, which is transmitted through the fiber with minimal loss. Consequently, its strain sensitivity remains as high as 90.73% after 50 m of transmission—substantially higher than that of conventional electrical sensing methods.

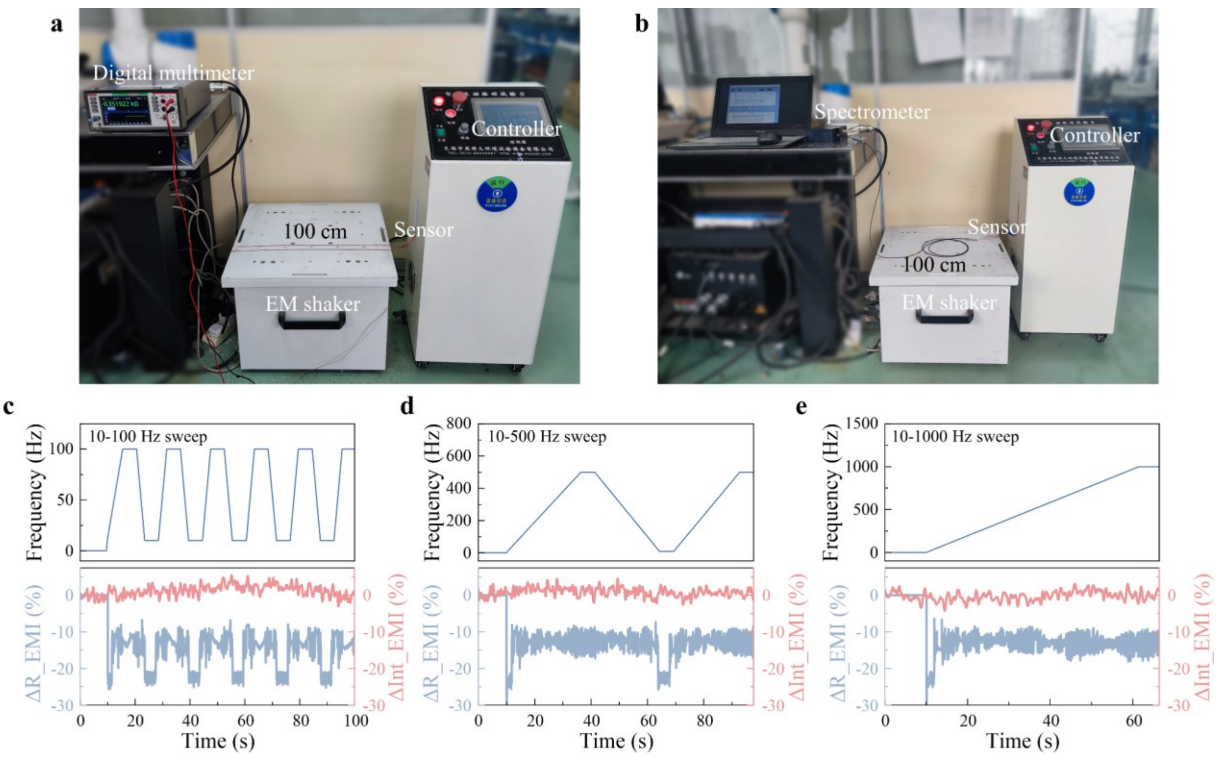

Figure S2. Comparison of EMI testing for purely electrical and electro-optical sensors. (a) Electromagnetic interference test for resistance-based electrical sensors. (b) Electromagnetic interference test for electro-optical sensors. (c-e) The response of both sensors to electromagnetic interference at different frequency sweeps: (c) 10–100 Hz, (d) 10–500 Hz, and (e) 10–1000 Hz. The blue and red curves represent the relative resistance change of the purely electrical sensor and the relative optical intensity change of the electro-optical sensor under electromagnetic interference, respectively.

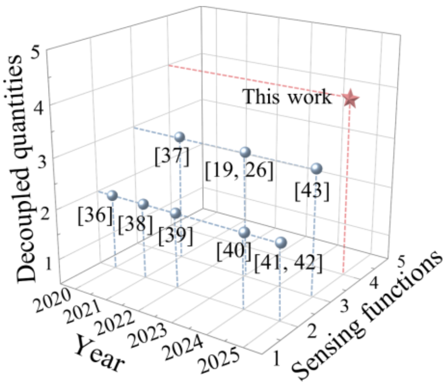

Figure S3. Comparison of the ESOT FiSensor with the functionalities of multimodal optical fiber sensors reported in recent years.

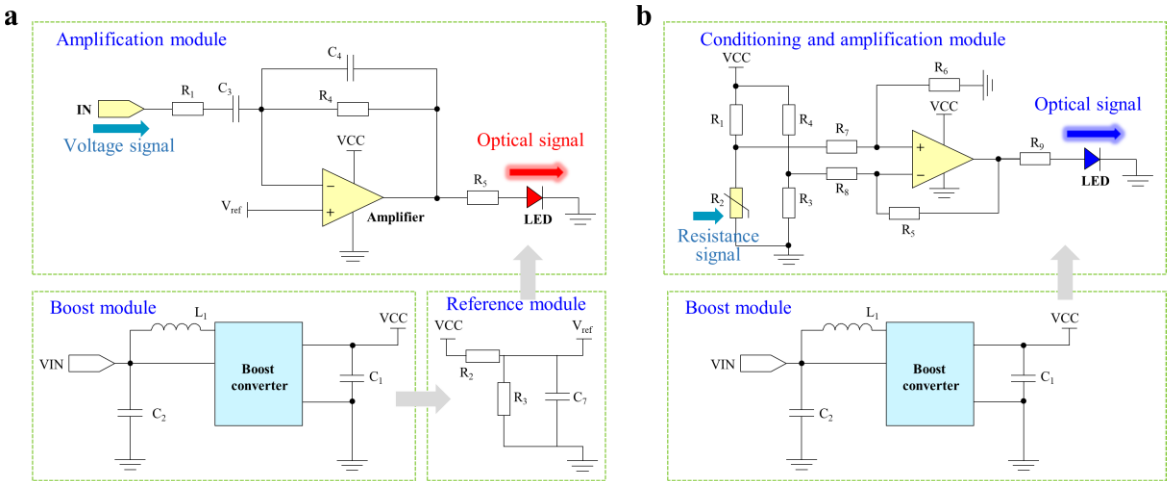

Figure S4. (a) Schematic of the piezoelectric-based signal conversion circuit. (b) Schematic of the resistance-based signal conversion circuit. By adjusting the external resistors R_1~R_7, the circuit can be adapted to sensors with different resistance variation ranges. It should be noted that for strain and pressure signals, R_1 in the schematic serves as the variable electrical sensor, while for temperature signals, R_2 functions as the resistance-type temperature sensor.

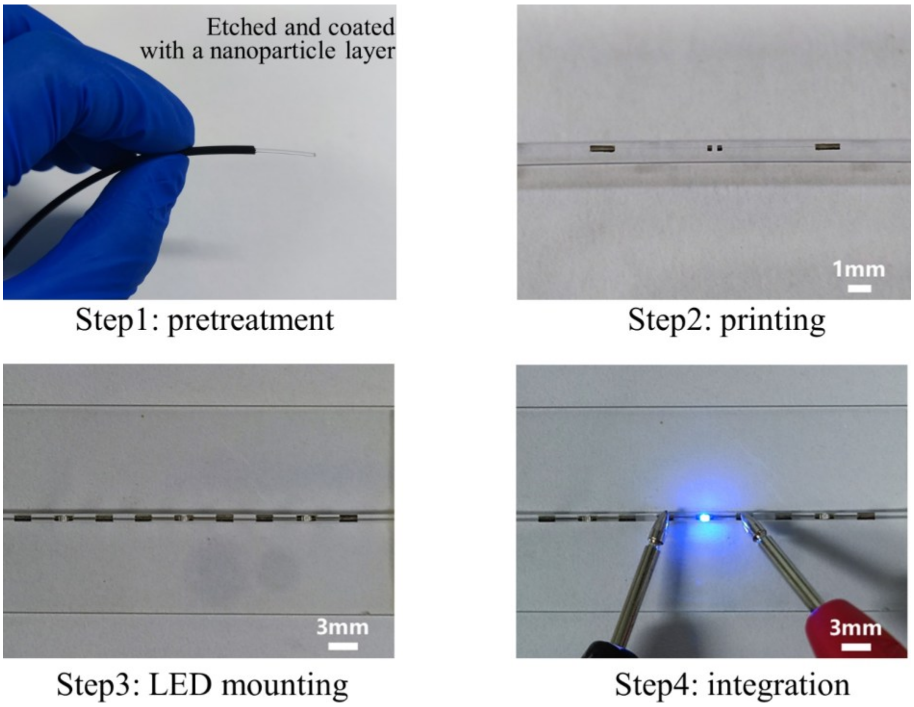

Figure S5. Fabrication process of the optical fiber surface circuit. First, Acetone was used as an etching agent to remove the cladding of the POF via wet etching. Then, a nanoparticle layer is deposited as a new cladding using dip coating method. Next, functional circuits are fabricated on the fiber surface through high-resolution EHD printing. Finally, an LED chip array is attached, enabling electro-optical conversion on the fiber surface.

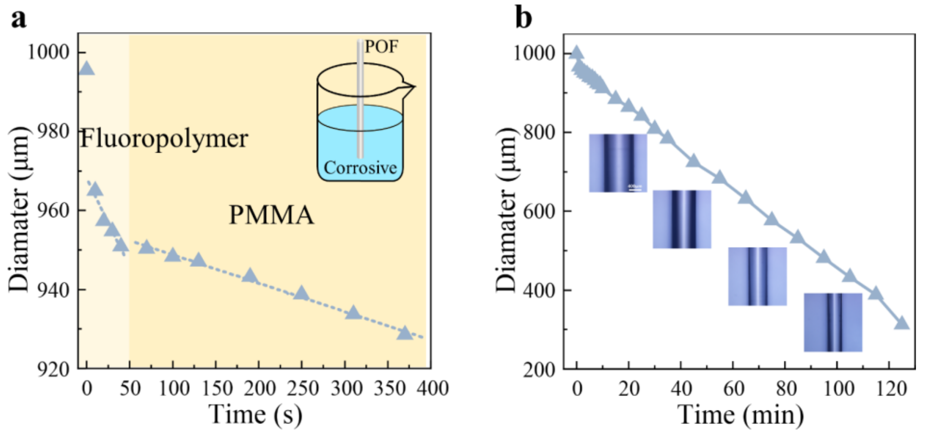

Figure S6. Diameter variation of POF with short-term (a) and long-term (b) treatment using the solution method. Most of the cladding is removed after approximately 50 seconds of acetone treatment. Due to slight variations in the cladding thickness of each POF, the fibers are treated for 15 minutes to completely remove the cladding, resulting in a diameter of approximately 900 μm, unless otherwise specified.

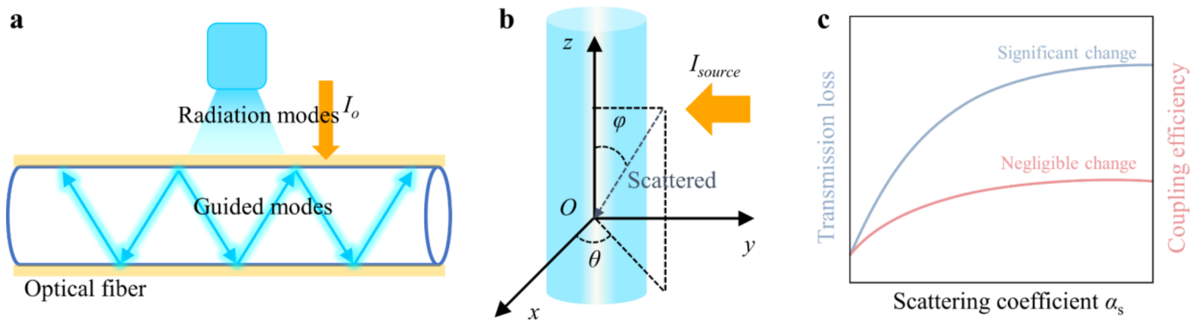

Figure S7. (a) Schematic diagram of the light path of the LED emission through the optical fiber surface. (b) A Cartesian coordinate system is established with the POF axis as the z-axis to analyze the coupling optical intensity from the LED into the optical fiber. (c) Effect of the scattering coefficient of the nanoparticle film on fiber transmission loss and side-coupling efficiency

A cartesian coordinate system is established with the fiber axis as the z-axis. Thus, I_0 is given by[1]:

$$I_0 = 2I_{\text{source}}\, C_{\text{sc}}\, \rho_a \int_{\theta_c}^{\pi} \int_0^{\pi} p(\theta,\varphi)\sin\theta\, \mathrm{d}\theta\, \mathrm{d}\varphi \tag{S3}$$

where I_{source} represents the optical intensity emitted from the LED, C_{sc} is the scattering cross-section of the nanoparticles ($C_{\text{sc}} = \alpha_s/\rho_v$, where ρ_v denotes the number density of nanoparticles per unit volume), ρ_a denotes the nanoparticle number density per unit area, and $p(\theta, \varphi)$ is the scattering phase function of a single nanoparticle. θ_c represents the critical scattering angle in the θ-direction. Lateral coupling consists of intrinsic coupling from LED light within the acceptance angle and additional coupling enhanced by nanoparticle scattering. The scattering-enhanced coupling model, expressed in **Equation S1**, predicts that the local coupling strength increases with the scattering coefficient within a certain range. However, the scattering coefficient has a limited effect on total lateral coupling, which is primarily governed by the angular divergence of the LED. Since scattering-enhanced coupling constitutes only a small fraction of the lateral coupling, its effect on optical signal regulation can be neglected. Instead, the primary focus should be on minimizing its impact on transmission loss, for example, by increasing the uniformity and consistency of the nanoparticle film.

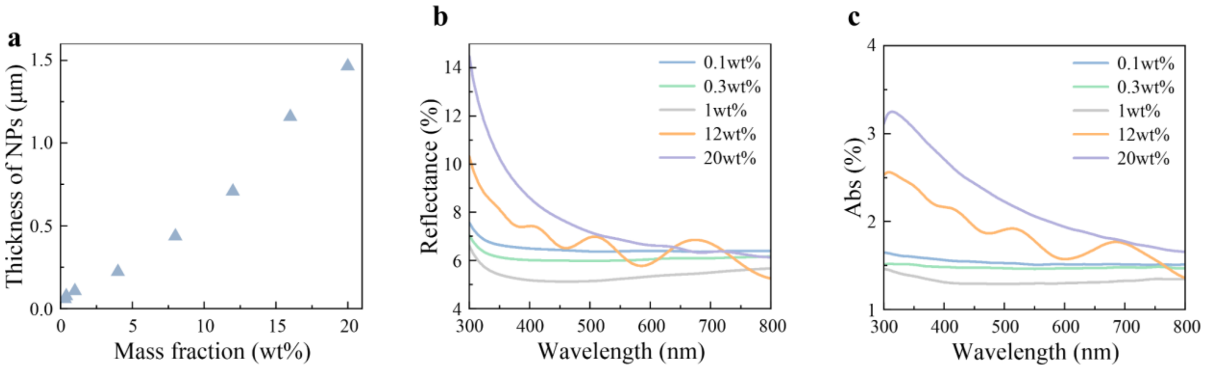

Figure S8. Characterization of the fundamental optical parameters of the aluminum oxide nanoparticle layer. Measured thickness (a), reflectance (b), and absorbance (c) of thin films prepared using nanoparticle dispersions with different mass fractions.

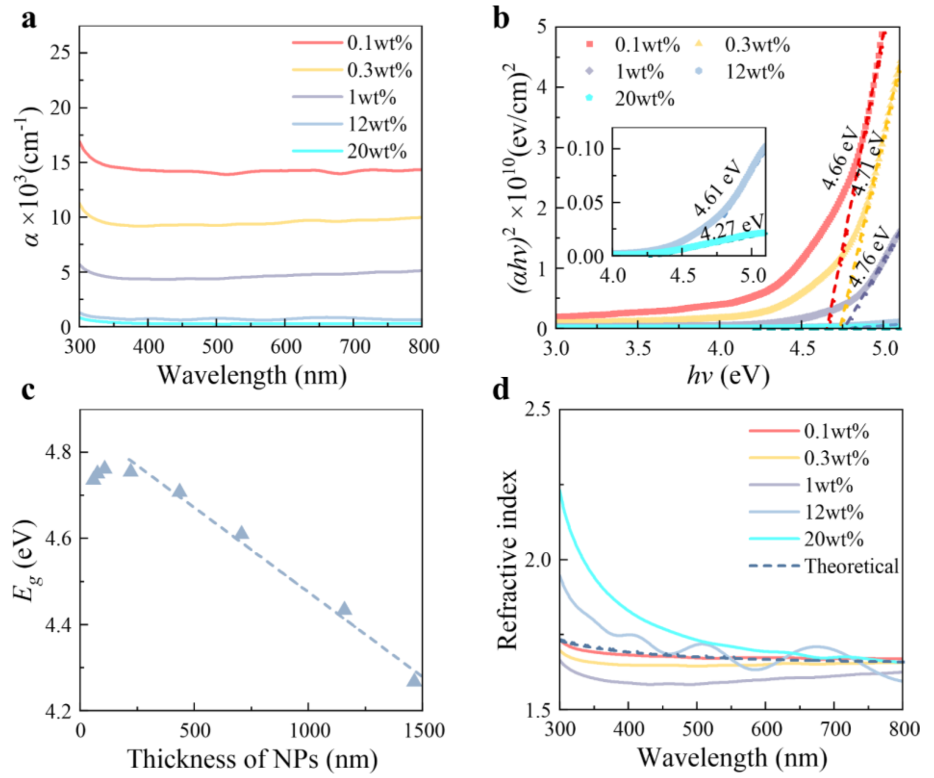

Figure S9. Experimentally measured and theoretically calculated optical properties of the aluminum oxide coating and its photon absorption capability. (a) Absorption coefficients of nanoparticle layers with different thicknesses. (b) Tauc plots used to determine the bandgaps of nanoparticle coatings with varying thicknesses, and (c) the corresponding bandgap values. (d) Comparison of experimental and theoretical refractive indices of aluminum oxide nanoparticle coatings as a function of thickness.

The relationship between the absorption coefficient and photon energy for different electronic transitions follows the Tauc model[2]:

$$(\alpha h v) = A(hv - E_g)^N \tag{S4}$$

where h is Planck constant, v is the photon frequency, hv is the energy of the incident photon, and αhv represents the absorbed photon energy per unit thickness. For direct allowed transitions, direct forbidden transitions, indirect allowed transitions, and indirect forbidden transitions, N takes specific values $N = \frac{1}{2}, \frac{3}{2}, 2, 3$, accordingly. Since aluminum oxide is a material with direct allowed transitions, we have $N = \frac{1}{2}$ .Based on the extinction coefficient (κ), the refractive index (n) was derived as follows:

$$n = \frac{1+R}{1-R} + \sqrt{\frac{4R}{(1-R)^2} - \kappa^2} \tag{S5}$$

The optical properties of films prepared from low- and high-concentration nanoparticle dispersions exhibit different trends, which may be attributed to variations in film density and defect levels. Pronounced interference effects are observed when the nanoparticle layer

thickness is a multiple of the wavelength, as seen in films prepared using a 12 wt% concentration.

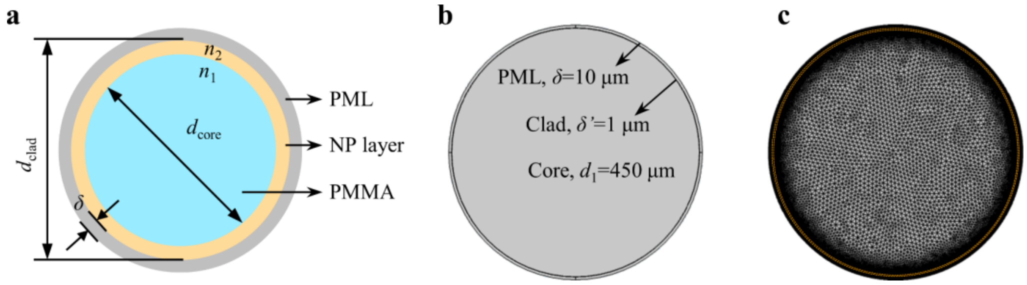

Figure S10. Simulation model configuration for transmission modes and loss in nc-POF. (a) Two-dimensional simulation cross-section and configuration parameters of the nanoparticle-coated POF (nc-POF). (b) Simulation calculation region. (c) Finite element mesh used in the model.

The transmission modes and confinement loss of the nc-POF were simulated using the Wave Optics Module in COMSOL Multiphysics. The fiber simulation structure was defined with three layers from the inside out: the core, the cladding, and a perfectly matched layer (PML). The cladding thickness was set to 1 μm, consistent with the typical thickness of films prepared using the dip-coating method. The PML was configured with a thickness of 10 μm to absorb electromagnetic waves at the interface and eliminate reflections. The transmission mode simulation was conducted based on the electric field distribution in the fiber cross-section. The core and cladding materials were defined as PMMA and aluminum oxide, respectively.

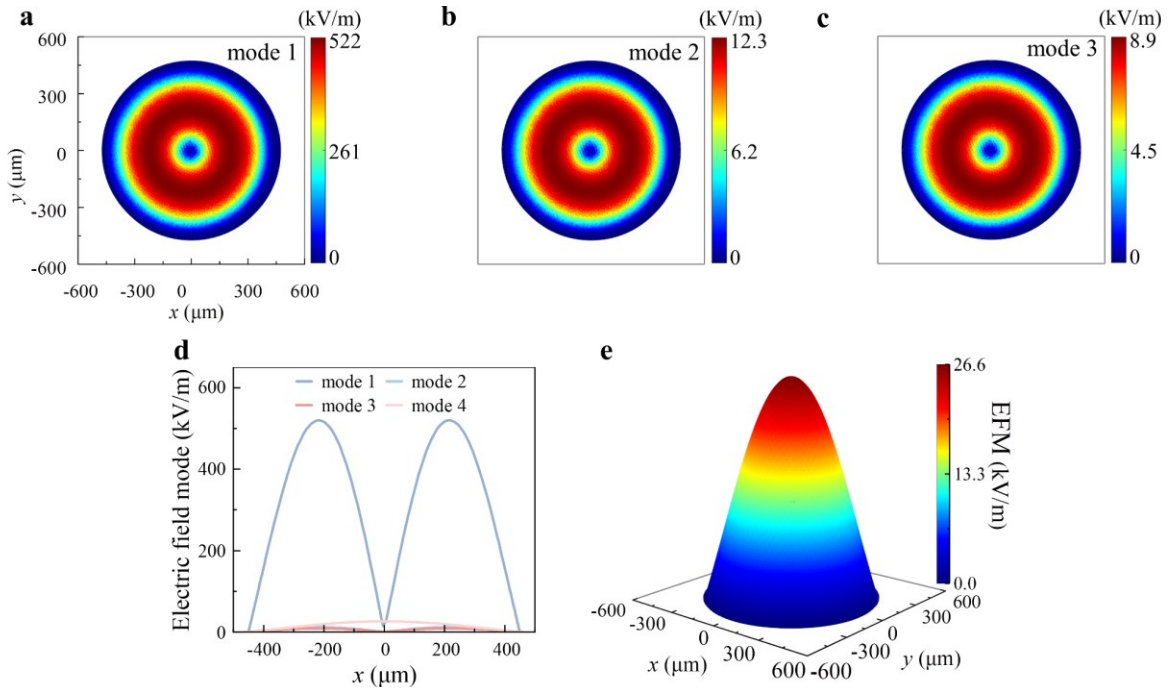

Figure S11. (a-c) Several high-order transmission modes in nc-POF. (d) Electric field distribution under different modes. Mode 4 corresponds to the fundamental mode shown in Fig. 2j, which is the primary transmission mode in the optical fiber. (e) The 3D height map of the typical fundamental mode distribution in the cross-section of nc-POF. (f) The variation of the confinement loss at a wavelength of 600 nm with respect to the core diameter.

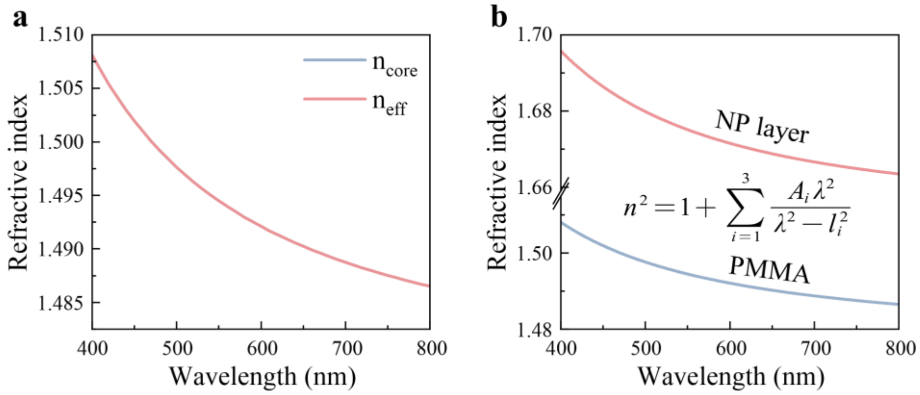

Figure S12. (a) Variation of the core refractive index and effective refractive index with wavelength in the fundamental mode simulation. (b) Dispersion curves describing the wavelength-dependent refractive indices of the cladding and core materials of nc-POF, formulated using a third-order oscillatory Sellmeier equation. Here, A_i represents the oscillation strength, l_i denotes the oscillation wavelength, and λ is the operating wavelength.

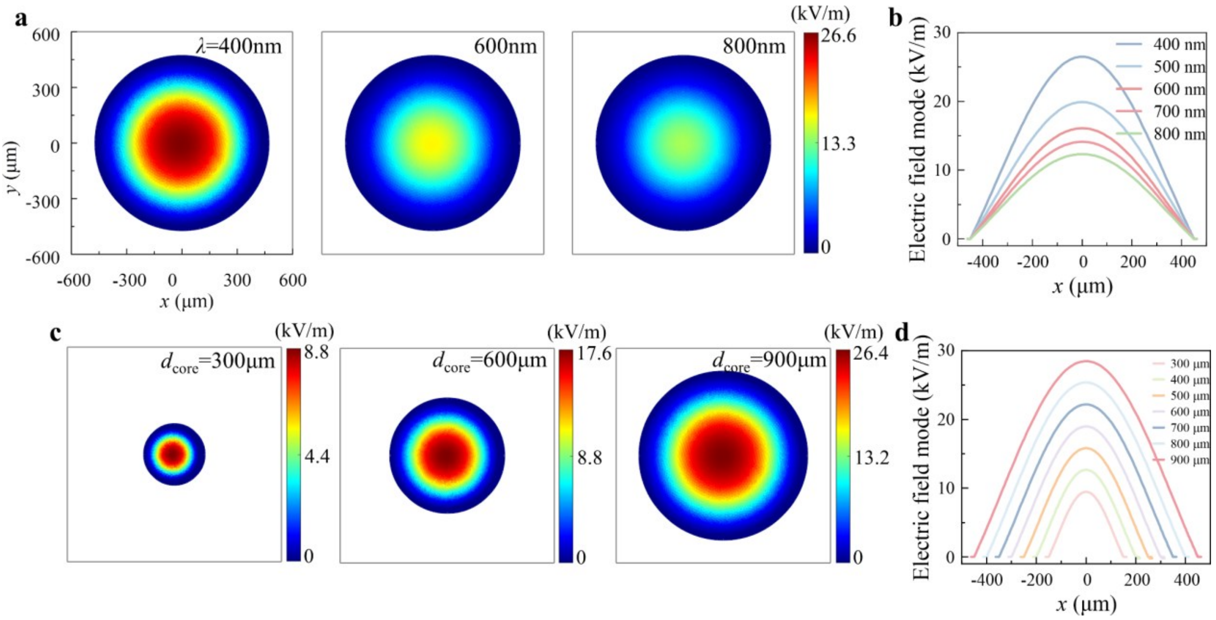

Figure S13. Electric field mode simulations of nc-POF under different operating wavelengths and core diameters. (a) Electric field distribution in nc-POF at different wavelengths. As the wavelength increases, the peak electric field magnitude gradually decreases. (b) Electric field mode along the x-axis within the nc-POF for different wavelengths. (c) Electric field distribution in nc-POF with different core diameters. As the core diameter increases, the peak electric field magnitude gradually increases. This indicates that maintaining a larger core diameter is preferable during the etching process of POF. (d) Electric field mode along the x-axis within the nc-POF for different core diameters.

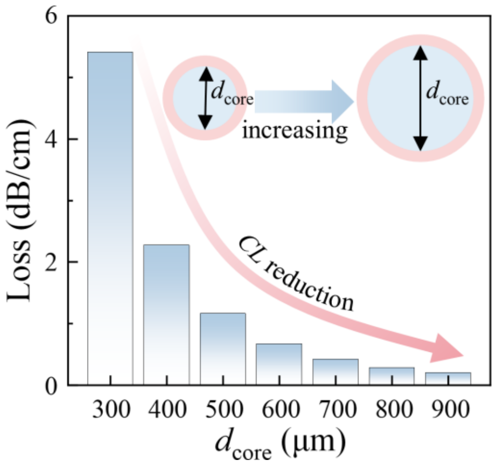

Figure S14. The variation of the confinement loss at a wavelength of 405 nm with respect to the core diameter.

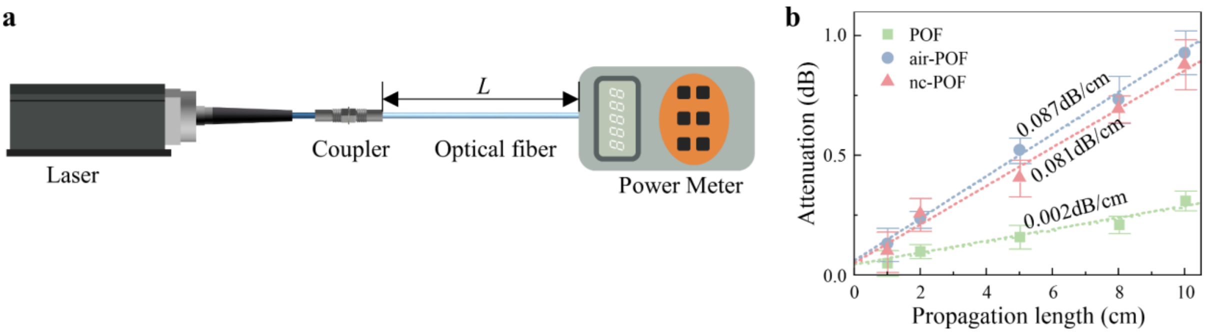

Figure S15. (a) Schematic diagram of the principle for testing the light power attenuation of POFs subjected to different treatments using the cutback method. The optical signal was generated by a 405 nm laser. (b) Transmission attenuations of three types of optical fibers measured using the cutback method: untreated POF, cladding-removed POF (air-POF), and cladding-removed POF with a nanoparticle coating (nc-POF).

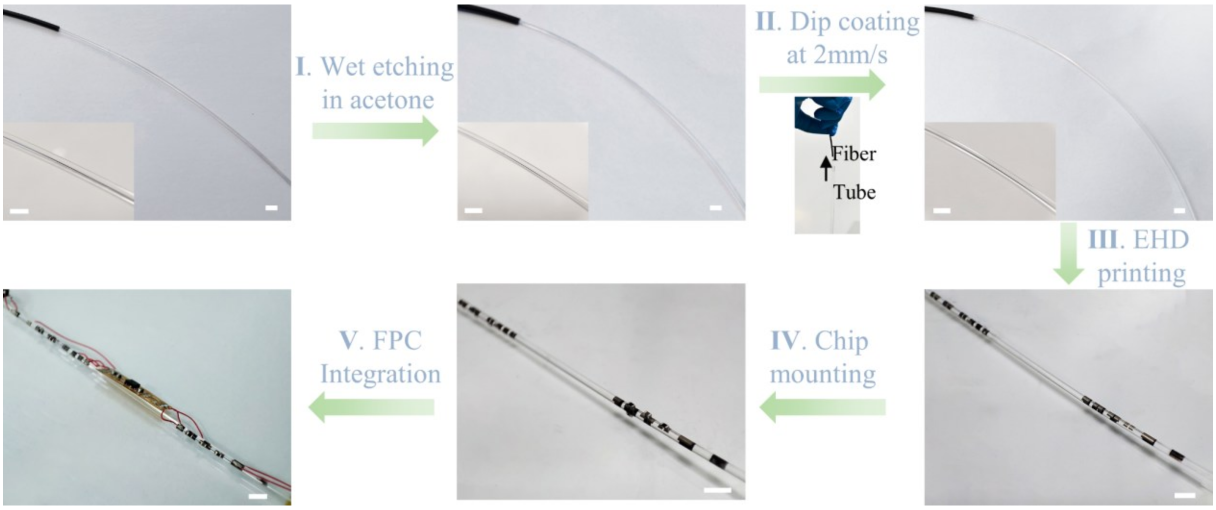

Figure S16. Fabrication process of the fiber-surface electro–optical conversion structure. The cladding of the original POF is first removed by wet etching to expose the bare core. A dielectric Al_2O_3 nanoparticle layer is then deposited on the core surface by a dip-coating method to enable stable and high-resolution EHD printing on the fiber. Circuit patterns are subsequently fabricated using microcylindrical EHD printing, followed by the mounting of chips such as resistors and LEDs. Finally, the printed circuit is integrated with an FPC to form a hybrid circuit. By interfacing with different front-end electrical sensors, the circuit can provide multiple sensing capabilities. It should be noted that the resistance values used in the conversion circuit must be adjusted to match the characteristics of different electrical sensors. Scale bar: 3 mm.

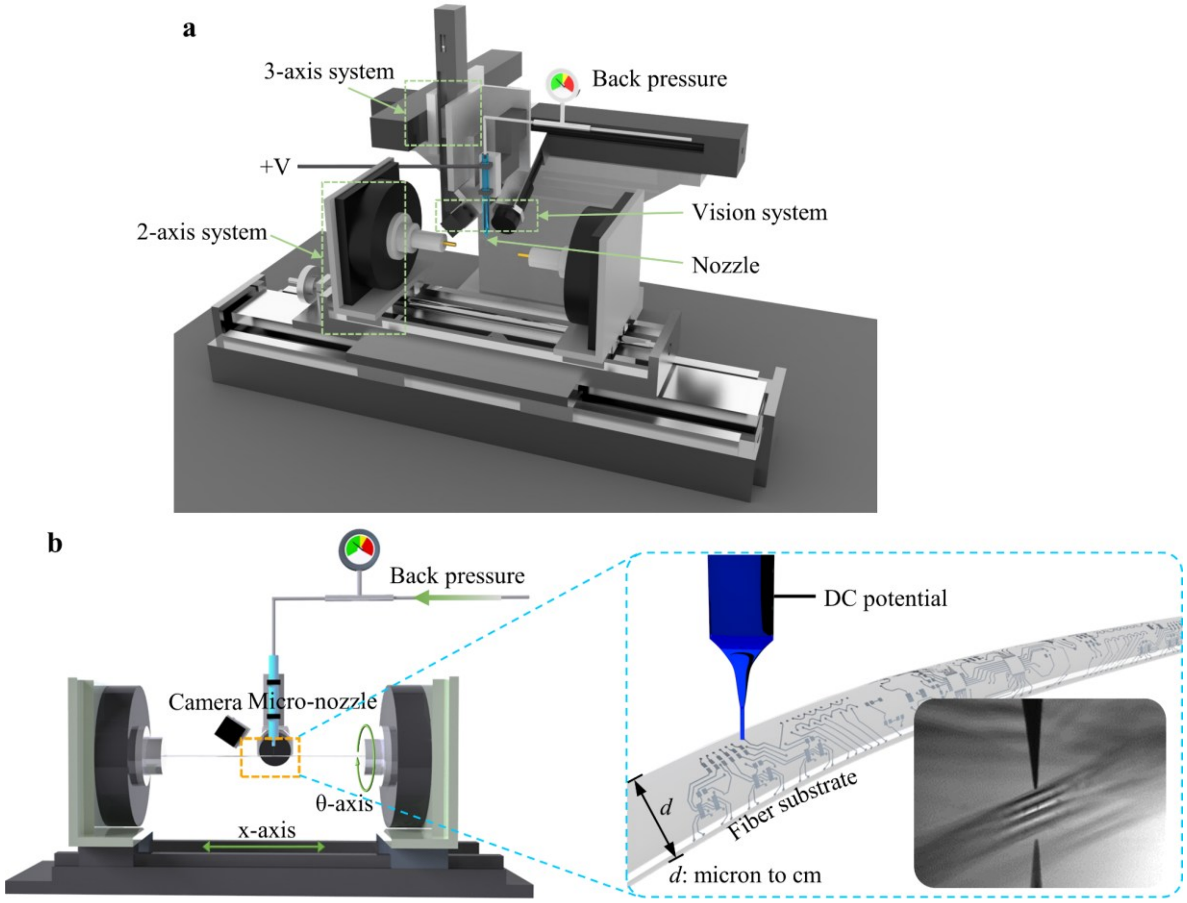

Figure S17. (a) Diagram of the micro-cylindrical EHD printing platform for high-resolution functional structure fabrication on fiber surfaces. (b) Principle and device for conformal EHD printing of functional structures on the fiber surface. A DC voltage is applied at the nozzle, while a three-axis motion platform and a high-precision fiber clamping mechanism enable high-resolution printing on the fiber surface.

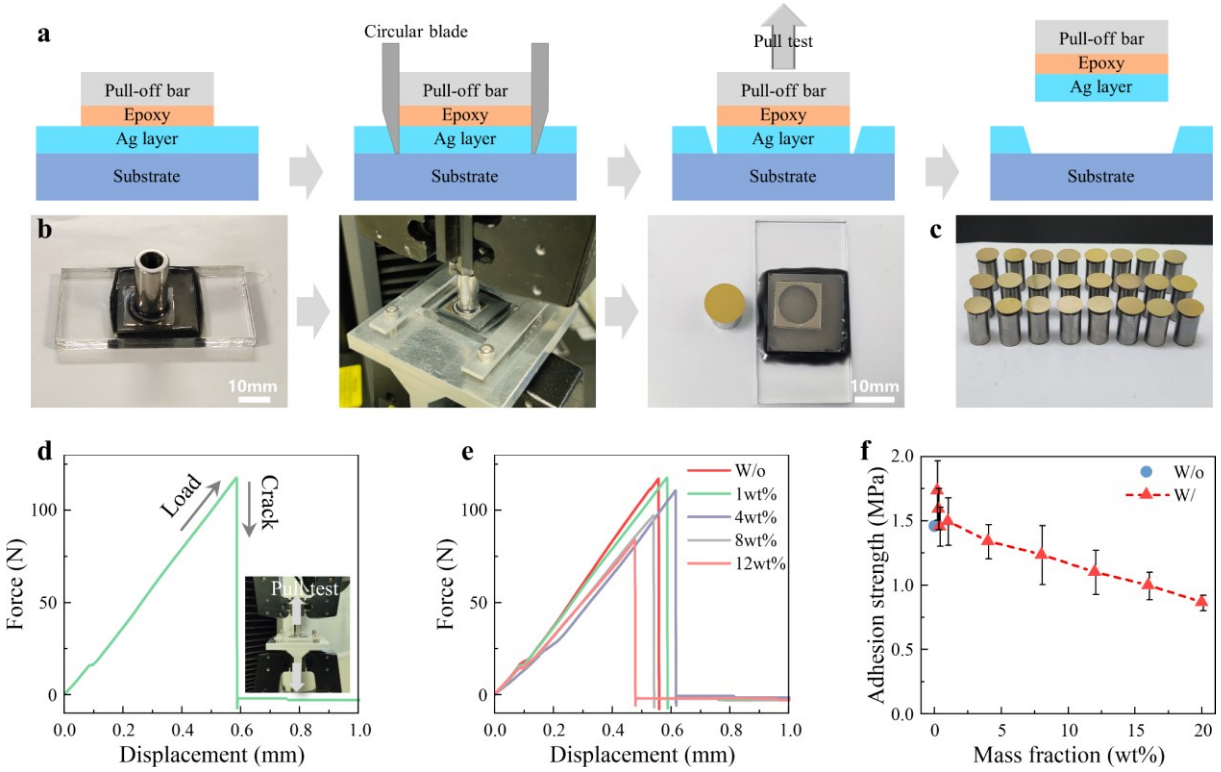

Figure S18. Effect of nanoparticle layers on the interfacial adhesion of printed structures. (a) Schematic of the interfacial adhesion test process, where a cured silver–epoxy composite layer is cut into a circular shape using a ring-shaped cutter and pulled vertically using a tensile tester until detachment occurs. The impact of nanoparticle coatings on the substrate surface is evaluated by comparing adhesion performance. (b) Photograph of the test setup. (c) Residual silver layer on the stud surface after the pull-off test. (d) Representative force–displacement curve during the detachment process. (e) Pull-off curves for substrates coated with nanoparticle layers of different thicknesses. (f) Interfacial adhesion strength as a function of nanoparticle layer thickness. When the thickness is below 100 nm, adhesion is comparable to that of the bare substrate. As the thickness increases, adhesion strength decreases. A nanoparticle dispersion with 4wt% is used in the experiments, yielding an adhesion strength approximately 80.2% of that on the bare substrate.

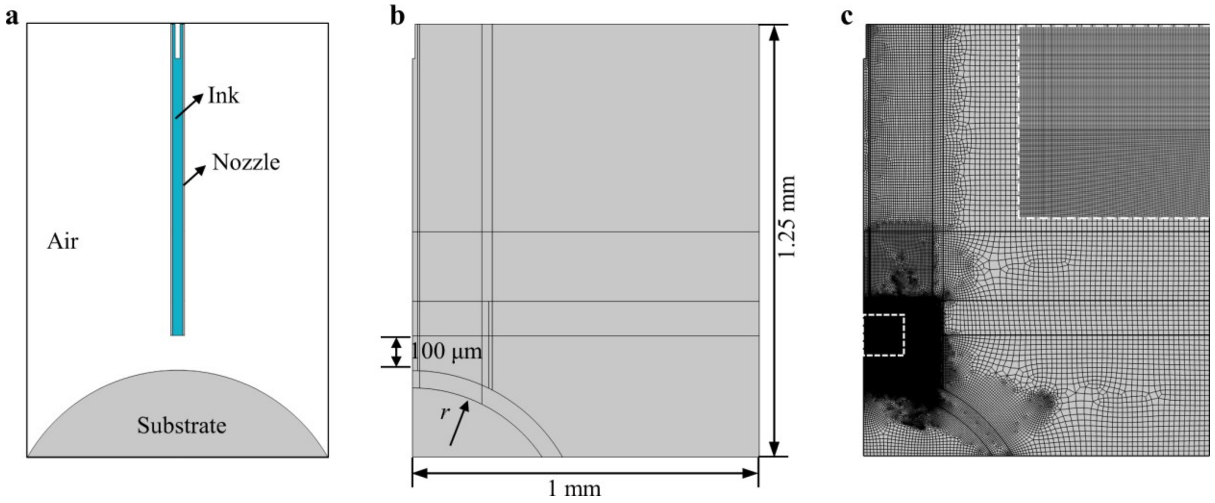

Figure S19. Finite element simulation of EHD printing on high-curvature micro-cylindrical surfaces. (a) Physical model, (b) two-dimensional axisymmetric model, and (c) computational domain with mesh discretization. The white box highlights a magnified view of the nozzle region. To enhance computational efficiency, a two-dimensional axisymmetric model is used to simulate the EHD printing process, simplifying the micro-cylindrical substrate to a spherical substrate with the same curvature.

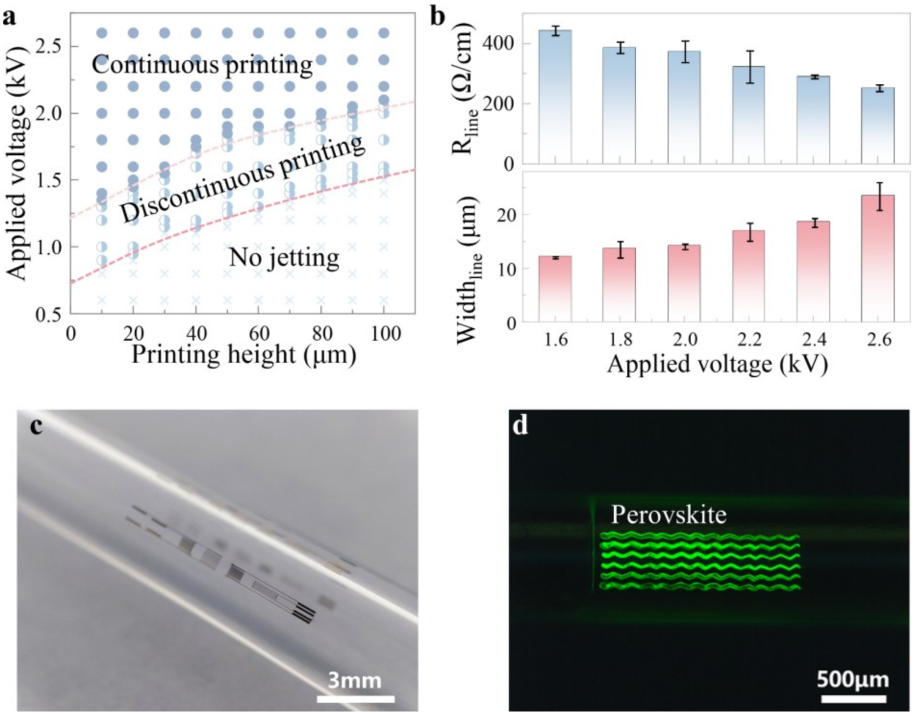

Figure. S20. Printing parameter optimization and validation. (a) Mapping of printing continuity versus printing height and applied voltage during EHD printing on a 1 mm diameter fiber. (b) Effect of applied voltage on the line width and resistance of the printed structures. (c) Optical image of a sensor array printed on the glass tube (*φ*~5 mm). (d) Perovskite fluorescent pattern printed on POF (*φ*~1 mm).

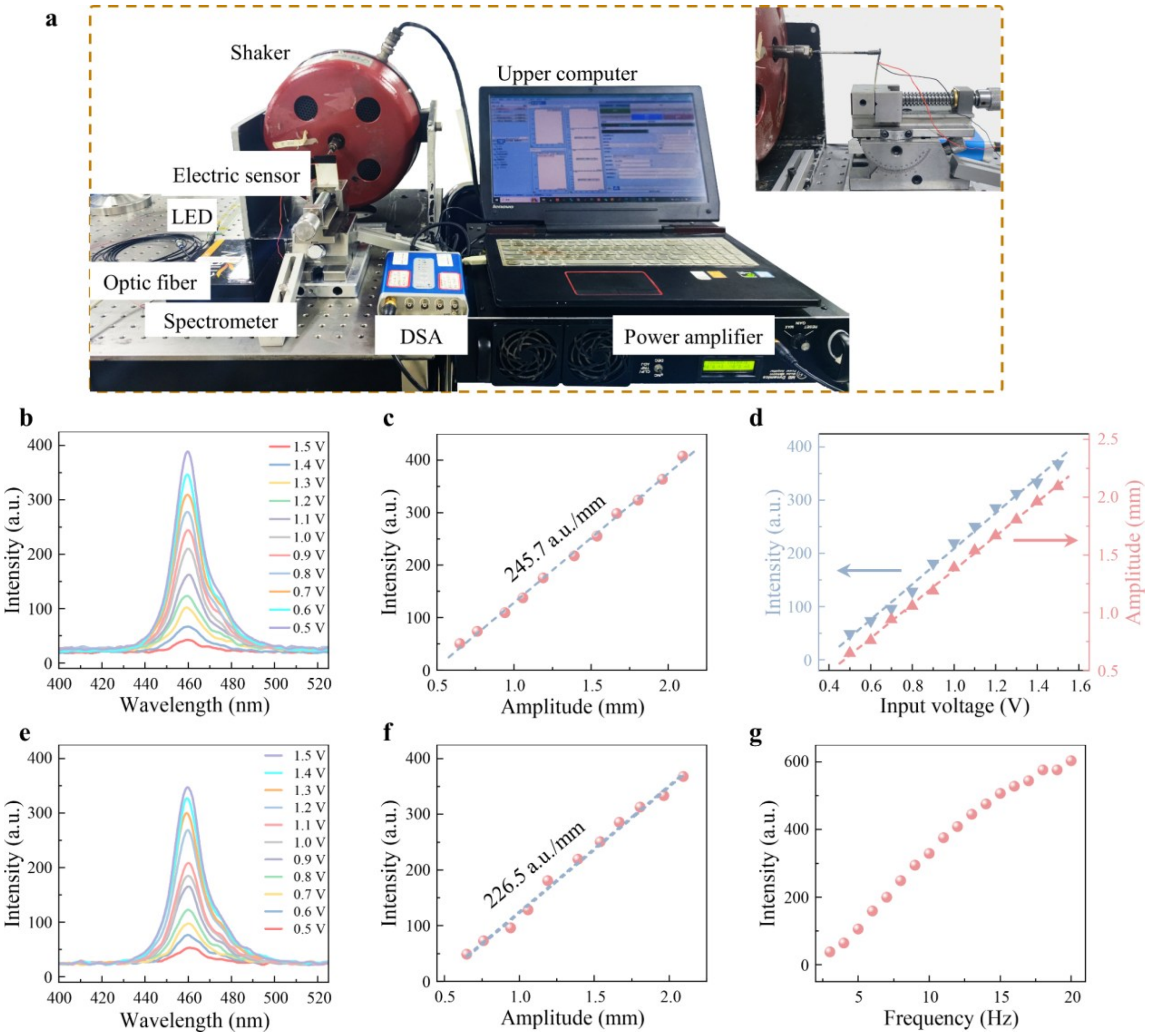

Figure S21. ESOT FiSensor for vibration signal monitoring. (a) The experimental setup for monitoring vibration signals using the ESOT FiSensor. The exciter oscillates at a controlled frequency and amplitude under signals provided by the dynamic signal analyzer (DSA) and power amplifier. The vibration signal is converted into an LED optical signal, which is transmitted via optical fiber to the spectrometer for detecting transmitted optical intensity. (b) Transmission spectra of the ESOT FiSensor under different exciter input voltages at a vibration frequency of 10 Hz. (c) Variation of detected optical intensity with exciter amplitude at 10 Hz. (d) Relationship between exciter input voltage, amplitude, and transmitted optical intensity at a vibration frequency of 20 Hz. (e) Transmission spectra of the ESOT FiSensor under different exciter input voltages at 20 Hz. (f) Dependence of detected optical intensity on exciter amplitude at 20 Hz. (g) Changes in transmitted optical intensity due to simultaneous variations in exciter amplitude when adjusting the frequency signal from the signal analyzer.

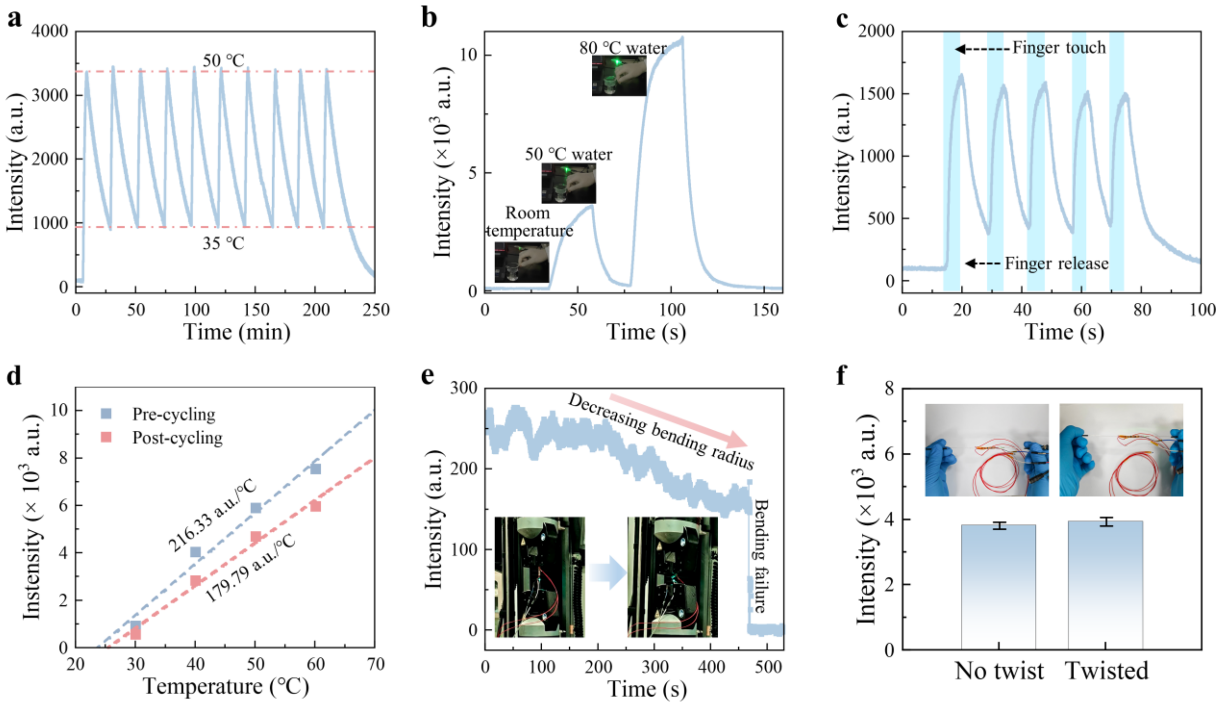

Figure S22. Temperature response of the ESOT FiSensor. (a) Variation in transmitted optical intensity over 10 heating-cooling cycles between 35 °C and 50 °C. (b) Intensity change when the sensor is brought near water cups at different temperatures. (c) Sensor response during finger touch and release, demonstrating its high sensitivity to temperature changes. (d) Temperature sensing sensitivity curves before and after the bending cycles. For clear comparison, four data points in the range of 30~60 °C, where the response is approximately linear, were fitted linearly. Bending of the ESOT FiSensor was induced by compressive loading. The transmitted optical intensity of the ESOT FiSensor was measured during 800 bending cycles at room temperature (~25 °C). The bending radius was approximately 72 mm. (e) Variation of the detected optical intensity as the ESOT FiSensor is bent from the initial state to failure. The final bending radius at failure is ~10 mm. (f) Comparison of the optical intensity in the untwisted state and under a 60° twist. The detected optical intensity after twisting shows almost no change compared with the initial state.

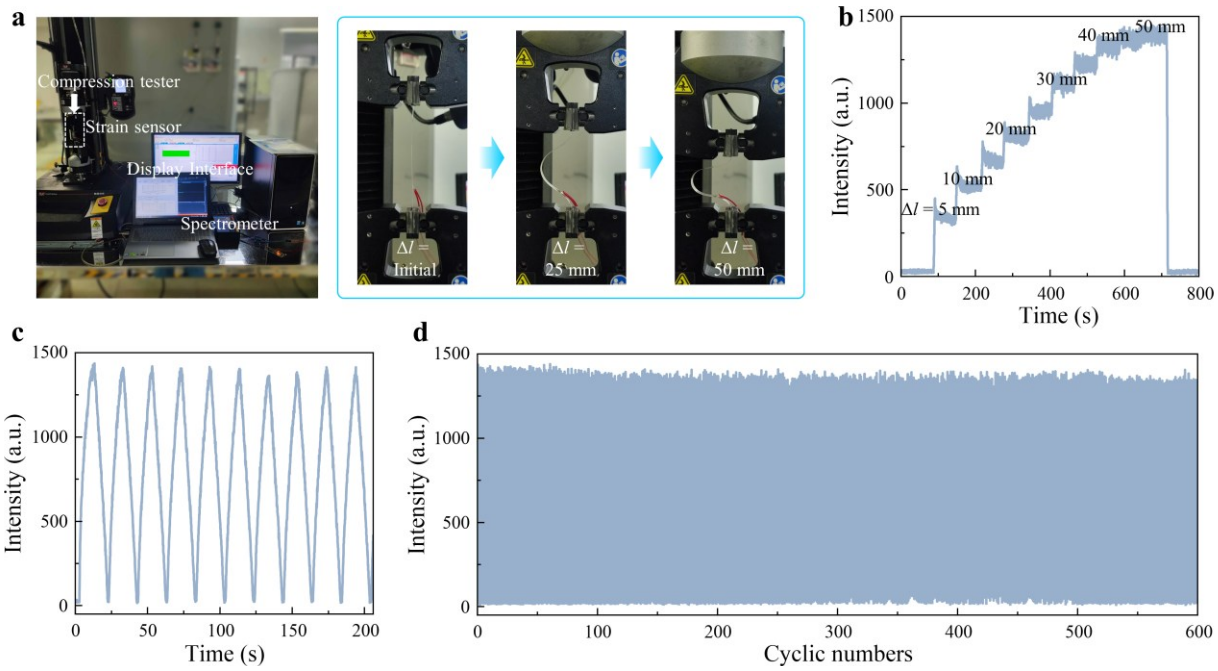

Figure S23. Strain signal detection via the ESOT FiSensor. (a) Configuration of the strain detection system based on the ESOT FiSensor (left). Different strain signals are generated by compressing varying displacements using a compression tester (right). (b) Transmitted optical intensity under varying compressive displacements, with a resistance-based strain sensor at the front end. Stable performance of the ESOT FiSensor over short-term (10 cycles, (c)) and long-term (600 cycles, (d))

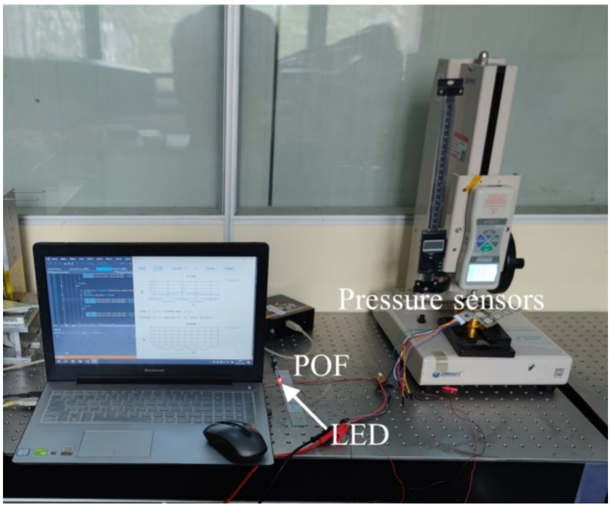

Figure S24. The measurement setup for pressure signal detection via an electro-optical sensor.

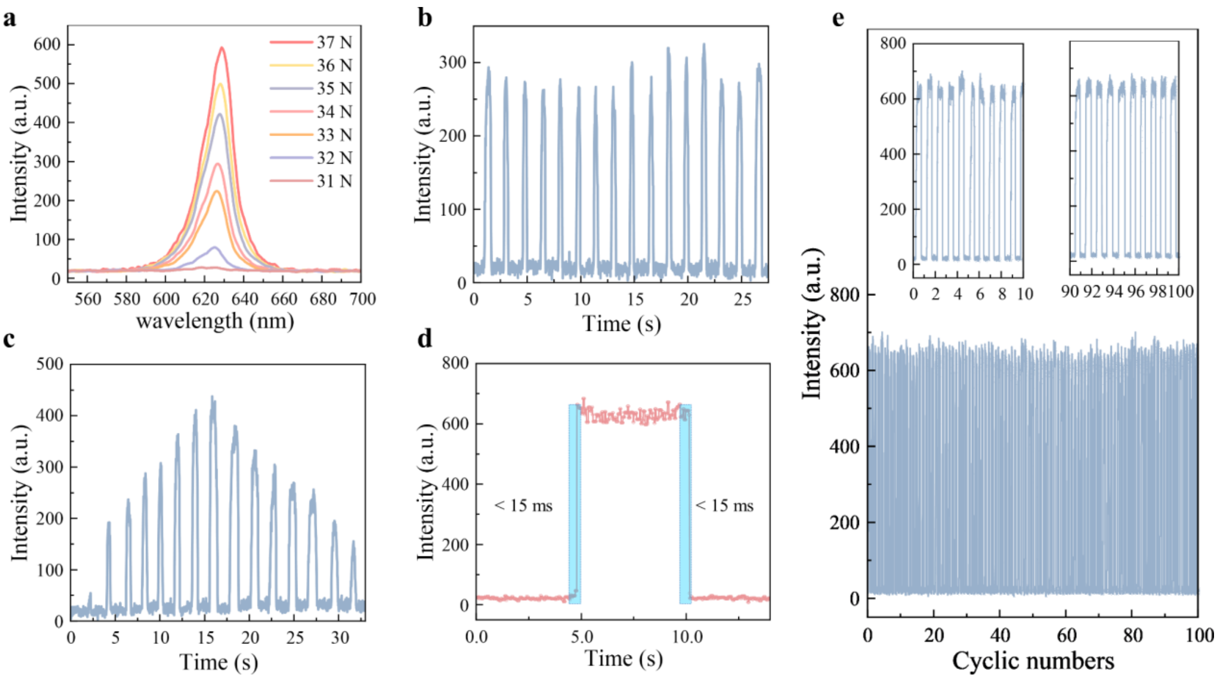

Figure S25. Pressure response of the ESOT FiSensor. (a) Transmission spectral response of the ESOT FiSensor under different applied pressures. (b) Response of the sensor to repeated finger pressing on the pressure sensor. (c) Electro-optical response under continuous pressure application with increasing and then decreasing pressure. (d) Rapid response of the pressure sensor. The ESOT FiSensor achieves a sub-15 ms response time, with further enhancement attainable through increased detection frequency of the spectrometer. (e) Response of the ESOT FiSensor under 100 consecutive finger presses.

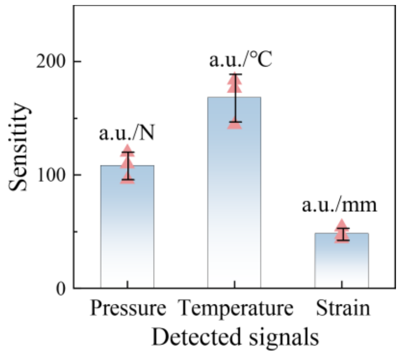

Figure S26. Device-to-device reproducibility of the ESOT FiSensor. Sensitivity statistics for pressure, temperature, and strain sensing obtained from three independently fabricated devices. Each data point represents an individual device. The horizontal line indicates the mean value and the error bars denote the standard deviation. The results demonstrate excellent device-to-device reproducibility.

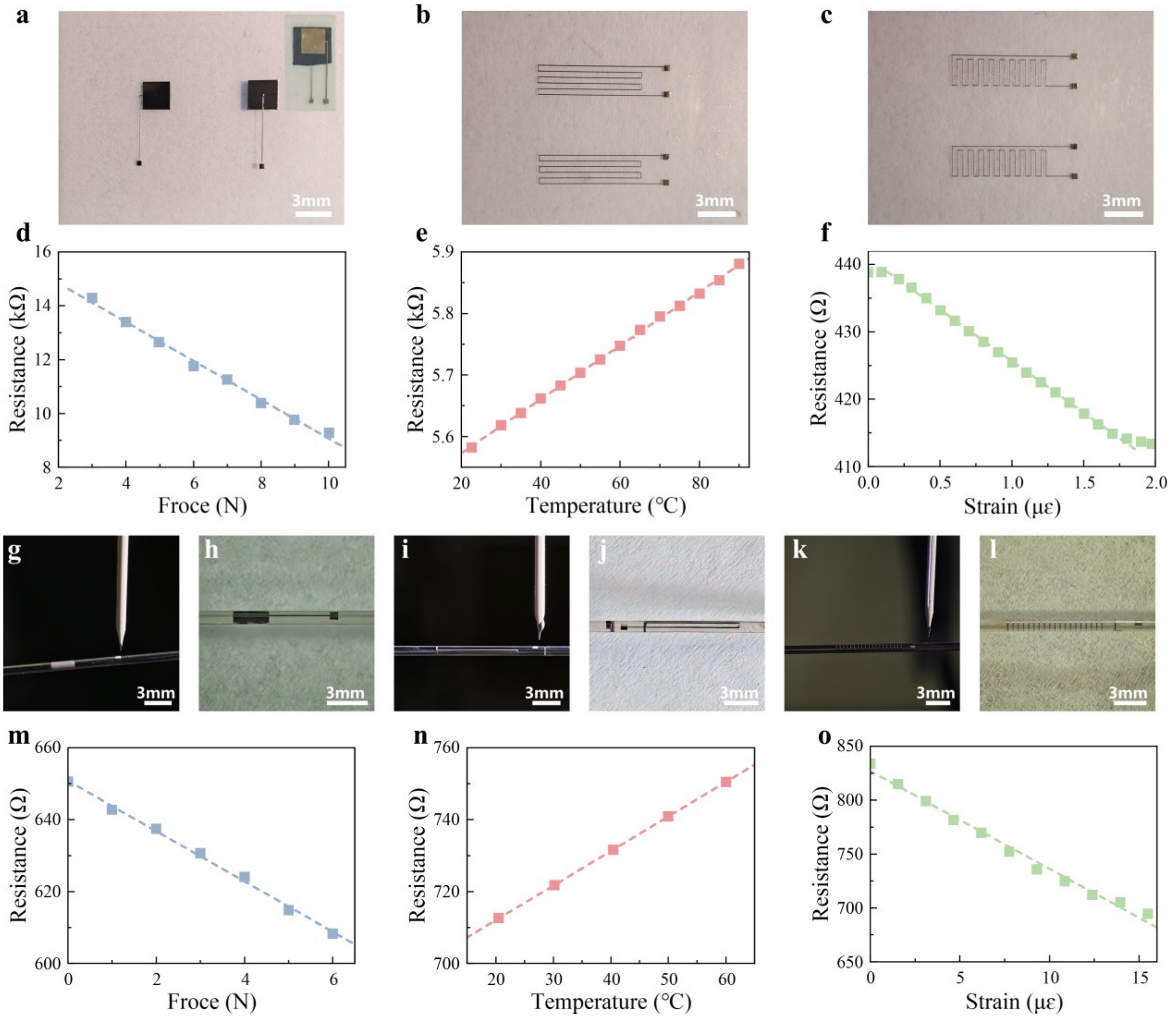

Figure S27. Fully printed electrical sensing units. (a–c) Printed pressure, temperature, and strain sensing units fabricated via e-Printing. The pressure sensor employs a silver-piezoresistive-silver multilayer structure. The temperature and strain sensors are fabricated using platinum and silver inks, respectively. These sensing units have compact dimensions at the millimeter scale. (d–f) Corresponding sensitivity curves for pressure, temperature, and strain sensing performance. All sensing elements exhibit linear resistance responses to external stimuli. Printing processes and completed images of pressure (g–h), temperature (i–j), and strain (k–l) sensing units on the fiber surface. (m–o) Corresponding sensitivity curves of fiber-conformal pressure, temperature, and strain sensing units.

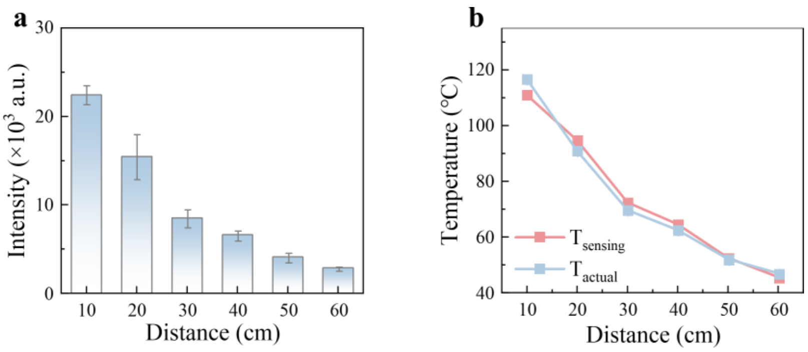

Figure S28. Optical intensity and measured temperature at different distances from the heat source. (a) Changes in optical intensity detected at different distances from the heat source. (b) Detected and actual temperatures as a function of distance from the heat source.

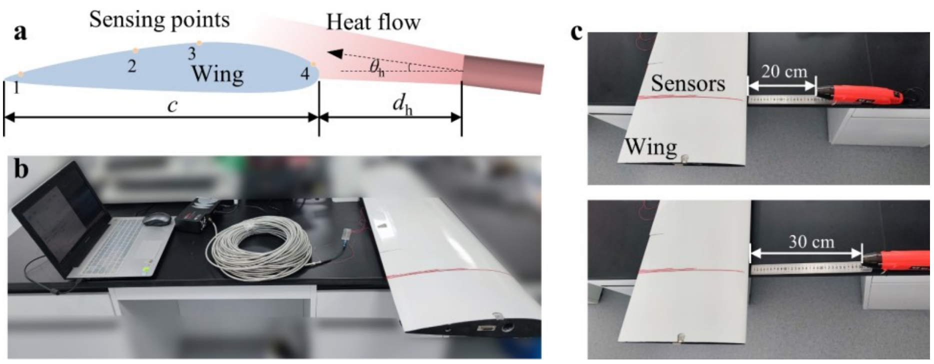

Figure S29. Distributed temperature sensing array for wing surface measurement. (a) Schematic of sensing points distribution along the chord direction of the wing and the heat flow direction. Because the heat gun used as the heat source has a supporting mount with finite height, the heat flow direction is tilted upward by approximately θ_h = 8°. (b) Photograph of the experimental setup. The resistance signals of the distributed temperature sensing array are converted into optical signals of LEDs on the fiber surface through the conversion circuit. (c) Testing procedure under heat flow applied at different distances dh from the wing.

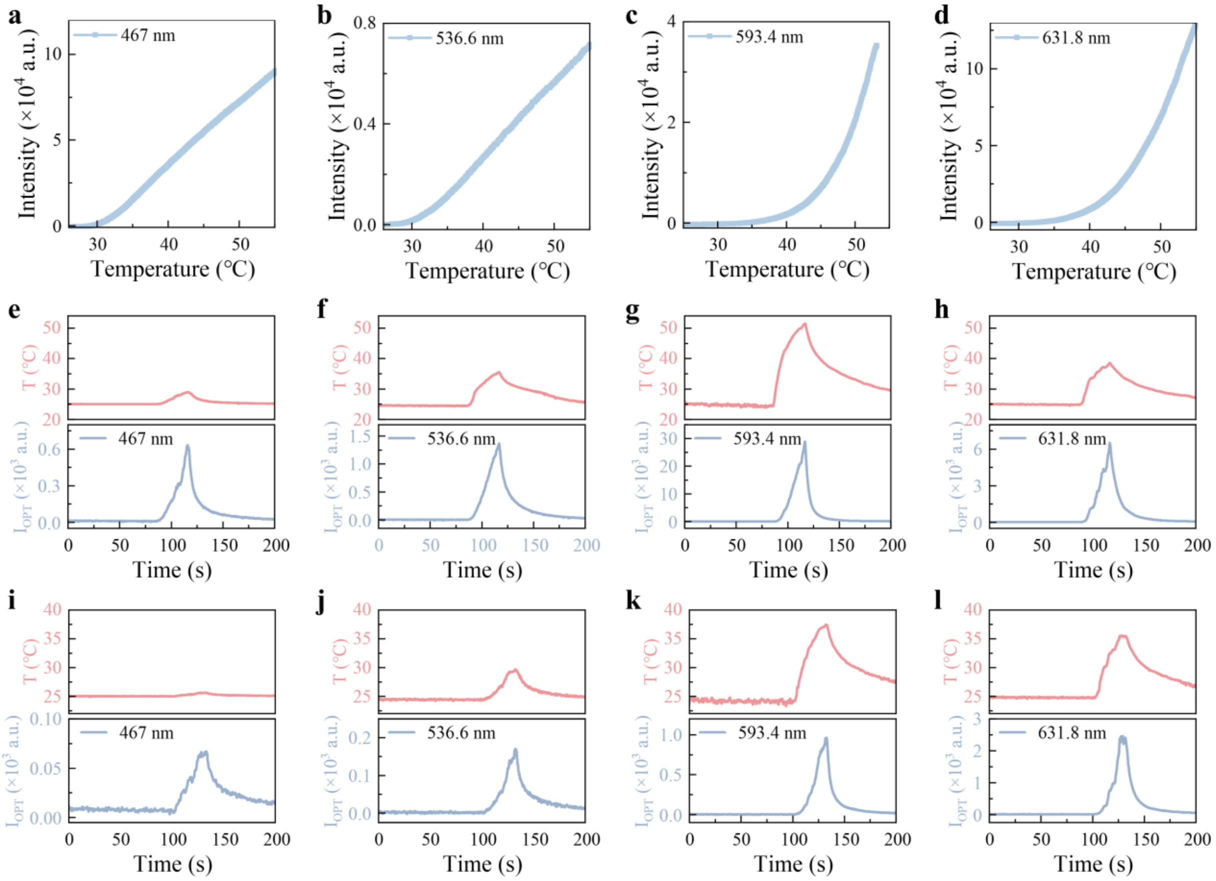

Figure S30. Calibration and testing of four temperature sensors. (a–d) Calibration curves of the four temperature sensors, showing signal intensity at different temperatures. Each detecting point is modulated at a distinct wavelength. Responses of the four sensors apart 20 cm (e–h) and 30 cm (i–l) away from the heat source after the wing is heated for 30s.

Since the operating voltage range varies among different LEDs, the resistance values in the circuit were finely adjusted, requiring recalibration of each temperature sensor. As shown in **Figure S30a–d**, the calibration data were used to back-calculate the detected temperature values at each position for comparison with reference measurements obtained from a commercial thermocouple thermometer.

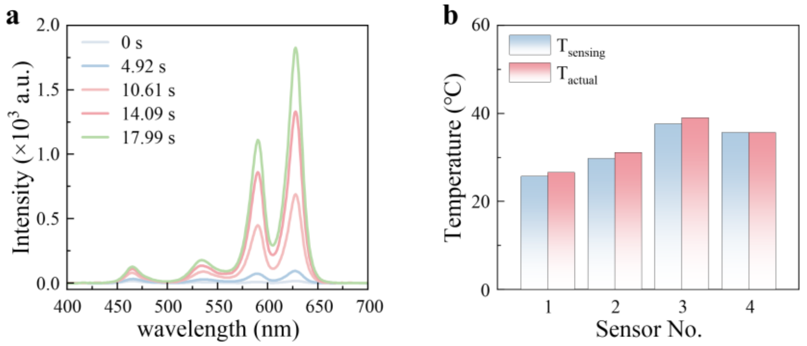

Figure S31. Comparison between detected and actual temperatures. (a) Spectral response at different times under continuous heating from a heat source placed at 30 cm. The four wavelength peaks correspond to sensing points at different positions. (b) Comparison between detected and actual temperatures after 30 s of heating at a distance of 30 cm.

$T_{sensing}$ and T_{actual} denote the temperature values measured by the sensor and by the standard thermocouple, respectively. The actual temperature distribution was influenced by the heat flux, which was emitted toward the wing surface at an angle. The temperature decreased rapidly from the central axis of the heat flux toward the edges, and also attenuated with propagation distance. Consequently, although position 4 was the first to encounter the heat flux, it was located near the periphery, where the carried thermal energy was lower than at position 3. Accordingly, the detected temperature at position 4 was generally lower than that at position 3 (Figure S31b).

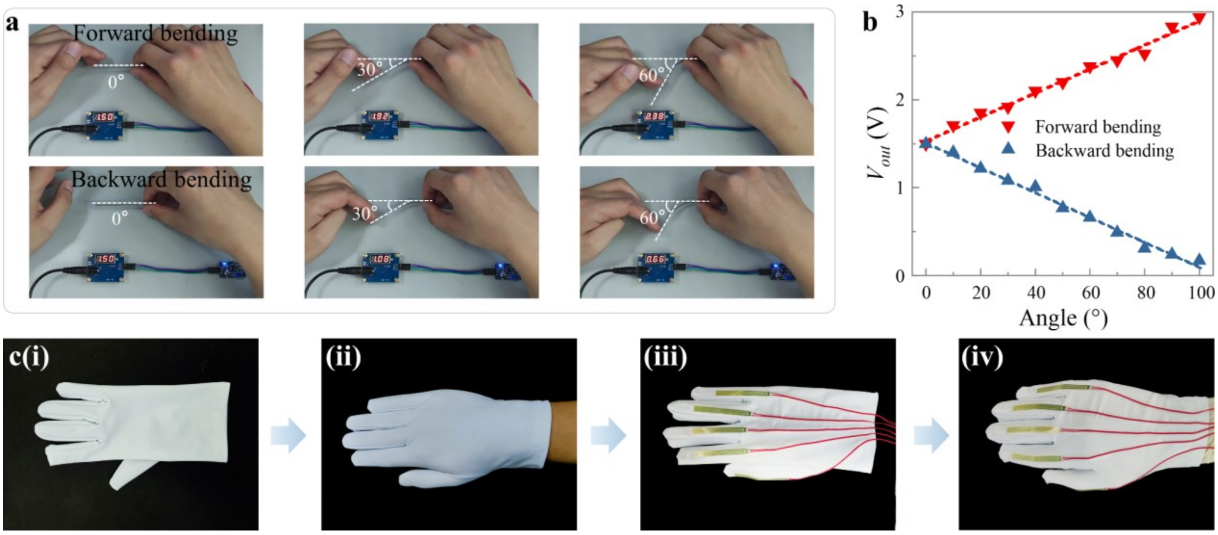

Figure S32. Characterization of the bending strain sensor and fabrication of the smart glove. (a) Variation in the output voltage out of the conversion circuit under forward and backward bending of the strain sensor. During the forward bending, the output voltage increases with the bending angle, While the output voltage shows an opposite trend during backward bending. (b) Variation in the output voltage of the conversion circuit under different forward and backward bending angles. (c) Fabrication process of the original smart glove (i) its wearable configuration (ii), Glove with attached strain sensors (iii) and its wearable configuration (iv).

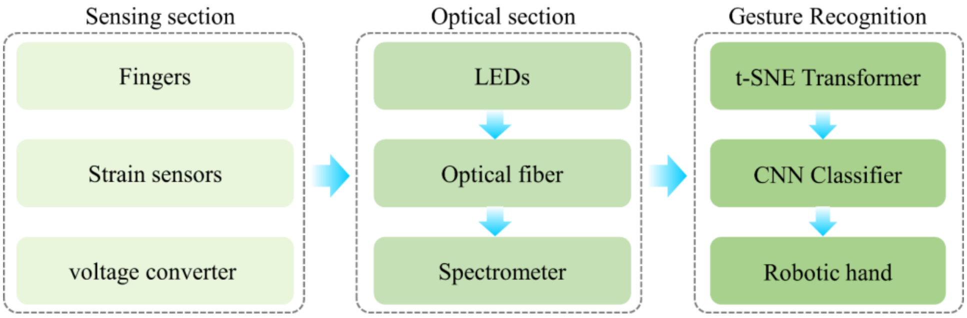

Figure S33. System architecture for gesture recognition using the ESOT FiSensor.

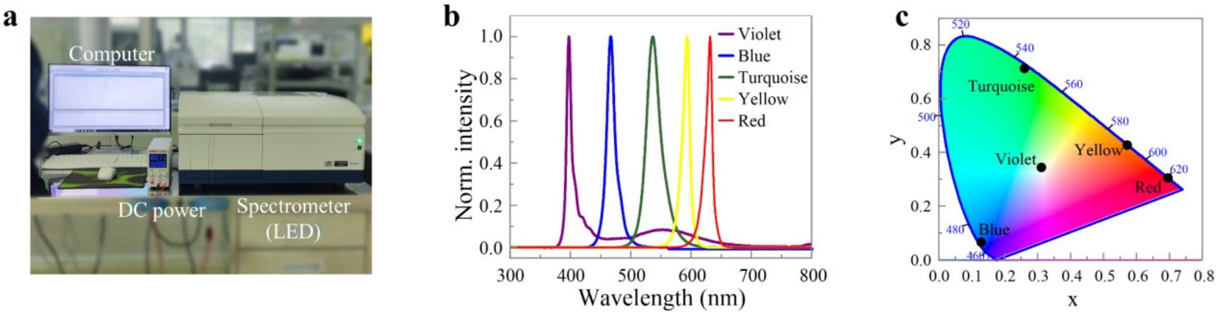

Figure S34. Fundamental spectral characterization of various LEDs. (a) Experimental setup for measuring LED emission spectra using a fluorescence spectrometer. (b) Normalized spectra of five LEDs used to monitor the five fingers and (c) their color coordinates in the CIE 1931 color space.

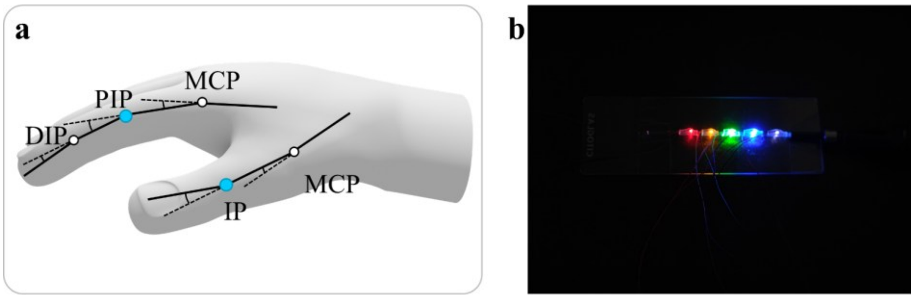

Figure S35. (a) Finger joint layout. (b) Optical images of five illuminated LEDs corresponding to the five fingers.

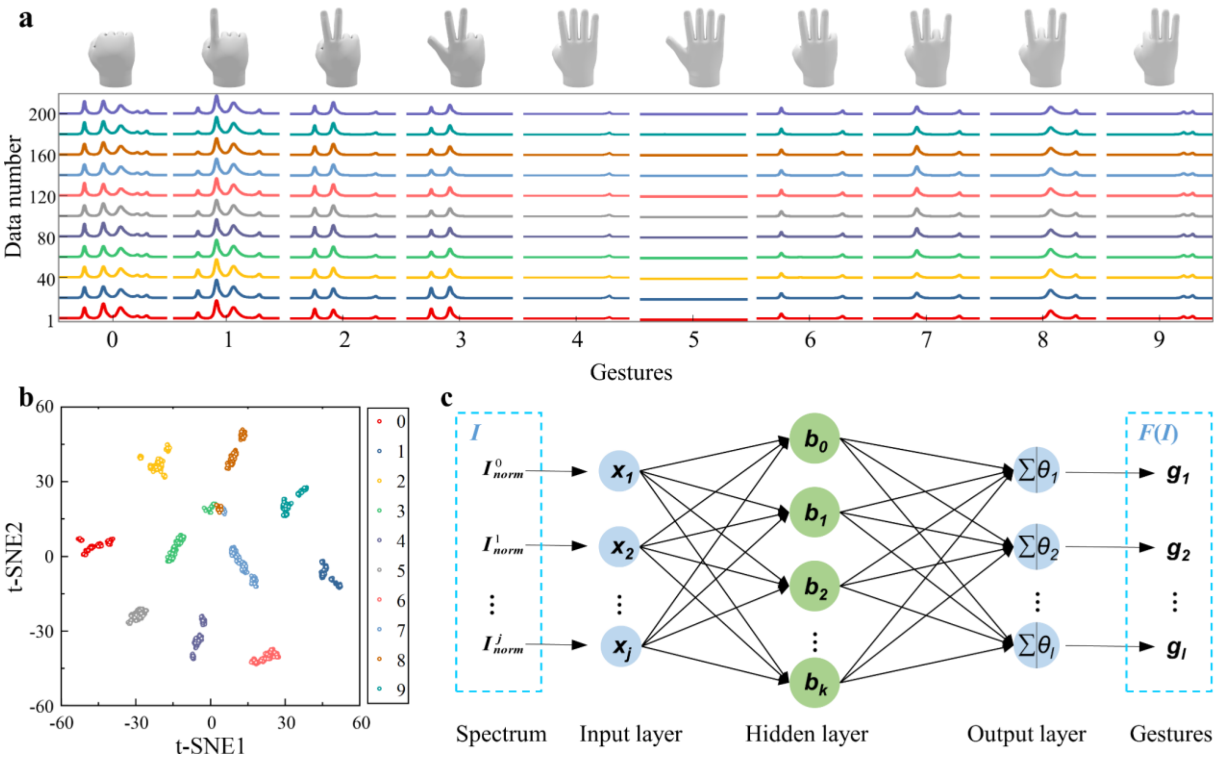

Figure S36. Acquisition and processing of gesture signals. (a) Response of the ESOT FiSensor to different gestures, with 200 data samples collected for each gesture. (b) Dimensionality reduction of spectral data from different hand gestures using the t-SNE algorithm. (c) Basic architecture of the ANN for gesture recognition.

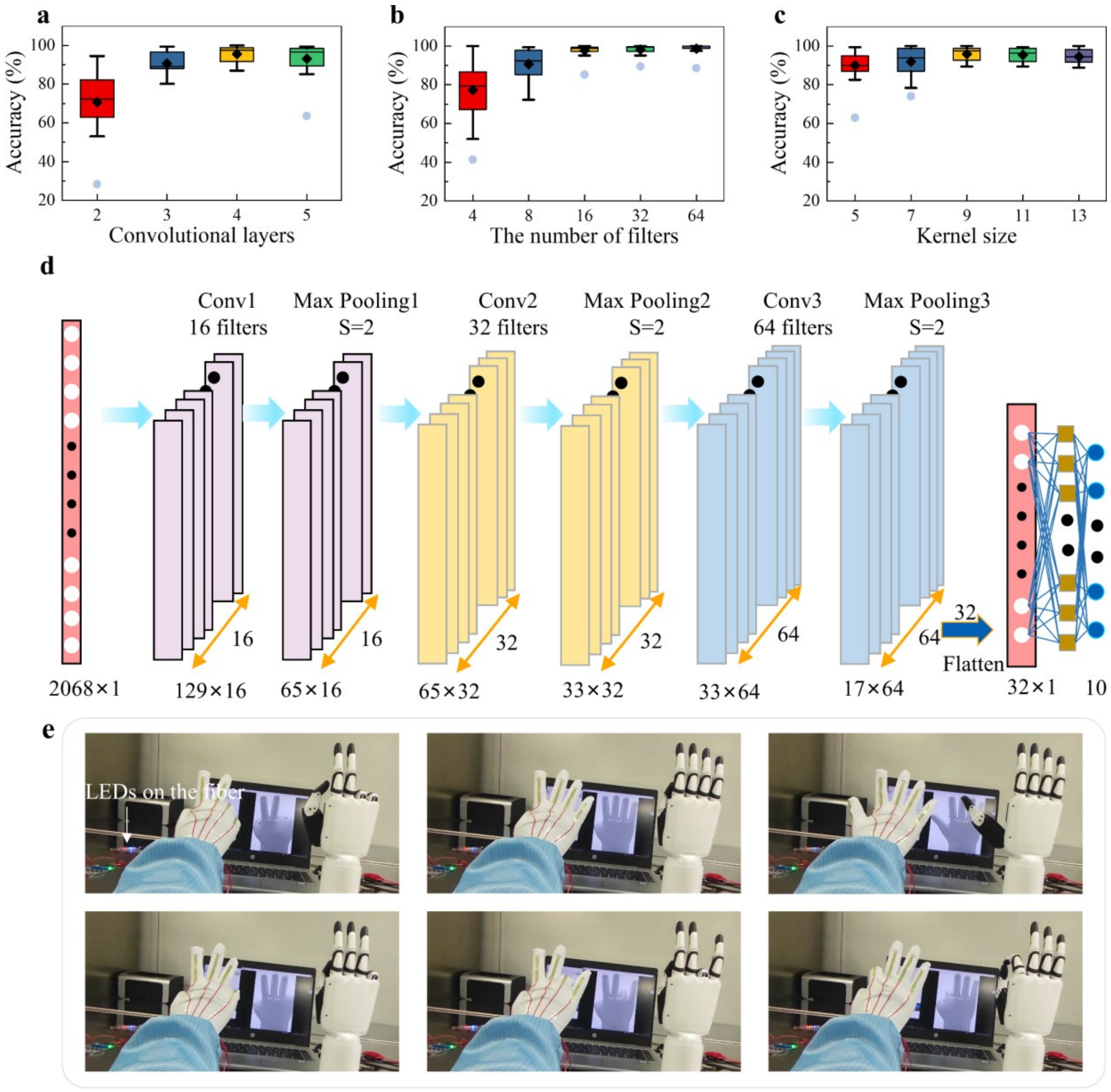

Figure S37. Gesture recognition and human–machine interaction demonstration. Neural network recognition accuracy under different (a) convolutional layer numbers, (b) filter numbers, and (c) kernel sizes. (d) Optimized ANN architecture for gesture recognition. (e) Photographs of real-time gesture recognition and robotic hand control using the ESOT FiSensor. The LED array on the fiber surface exhibits distinct emission spectra under different hand gestures. By analyzing the spectral information, an artificial neural network can be trained to facilitate human-machine interaction.

To validate the capability of the strain sensors in distinguishing different finger bending angles, the output voltage variations of the voltage conversion circuit were first measured under different bending angles (Figure S32a). The voltage exhibited opposite trends for forward and backward bending, indicating that the sensors can detect deformations in both directions. A clear linear relationship was observed between the bending angle and the output voltage in both directions (Figure S32b), establishing the foundation for strain-based electro-optical sensing. Five strain sensors were integrated into the finger sections of a smart glove to detect hand

gesture information (Figure S32c). The system architecture for gesture recognition with electro-optical sensing is illustrated in Figure S33. The minute resistance variations of the five strain sensors were converted into voltage signals by the voltage conversion circuit and then used to drive individual LEDs. The transmitted optical intensity at different wavelengths was demodulated using a spectrometer. Then the t-distributed stochastic neighbor embedding (t-SNE) algorithm was employed for dimensionality reduction and feature extraction, preserving key characteristics while minimizing data complexity. Finally, a CNN was trained to classify specific hand gestures. Building on this, the system can also be utilized for robotic arm control to enable remote operation.

For remote electro-optical sensing, five commercially available LED chips with different emission wavelengths were tested, and their spectral characteristics were analyzed to obtain their chromaticity coordinates. To minimize signal crosstalk, five LEDs with distinct colors (violet, blue, cyan-green, yellow, and red) were selected and integrated with the strain sensors on each finger to enable distributed sensing (Figure S34). Although the human finger consists of the metacarpophalangeal joint (MCP), proximal interphalangeal joint (PIP), distal interphalangeal joint (DIP), and interphalangeal joint (IP) (Figure S35a), the key joint for gesture recognition is the IPP. Therefore, attaching the strain sensor conformally to the PIP joint is sufficient to effectively detect angular variations in the finger joints (Figure S35b). Gesture training and recognition for gestures 0-9 were conducted by an experimenter wearing the smart glove (Figure S36a). The spectral data for each gesture were demodulated using a spectrometer, and dimensionality reduction was performed using the t-SNE algorithm (Figure S36b). The data were then used for neural network training and testing. The architecture of the CNN for gesture recognition is shown in Figure S36c. The effects of different parameters, such as the convolutional layers, filters, and kernel size, on gesture recognition accuracy were analyzed (Figure S37a–c), and the optimal neural network framework was determined (Figure S37d). After importing the normalized spectral data Inorm, the recognition probabilities for the gestures were obtained, reaching 98.15% (Figure S37e). Building on this foundation, the system can further enable posture control of a robotic hand. Several representative gestures were selected to validate its effectiveness in human–machine interaction (Figure S37f). It is observed that the LED emission patterns vary across different gestures. After neural network training, the system can accurately recognize gestures in real time, displaying them on a screen and precisely controlling the robotic hand to replicate human movements.

References

1. Fallah H, Chaudhari M, Bora T, et al. Demonstration of side coupling to cladding modes through zinc oxide nanorods grown on multimode optical fiber. *Opt. Lett.* 2013, 38, 3620-22.
2. Yu Z, Yu J, Zhang Y, et al. Microstructural and nonlinear optical properties of quaternary gallium-titanium-zinc-oxide transparent conductive semiconductor thin films. *Funct. Mater. Lett.* 2023, 16, 2351018.
